# Impact of COVID‐19 and other infectious conditions requiring isolation on the provision of and adaptations to fundamental nursing care in hospital in terms of overall patient experience, care quality, functional ability, and treatment outcomes: systematic review

**DOI:** 10.1111/jan.15047

**Published:** 2021-09-23

**Authors:** Rebecca Whear, Rebecca A. Abbott, Alison Bethel, David A. Richards, Ruth Garside, Emma Cockcroft, Heather Iles‐Smith, Pip A. Logan, Ann Marie Rafferty, Maggie Shepherd, Holly V. R. Sugg, Anne Marie Russell, Susanne Cruickshank, Susannah Tooze, GJ Melendez‐Torres, Jo Thompson Coon

**Affiliations:** ^1^ College of Medicine and Health University of Exeter Exeter UK; ^2^ The National Institute for Health Research (NIHR) Applied Research Collaboration (ARC) South West Peninsula (PenARC) Exeter UK; ^3^ Department of Health and Caring Sciences Western Norway University of Applied Sciences Bergen Norway; ^4^ School of Health and Society University of Salford Salford UK; ^5^ Northern Care Alliance NHS Group Salford UK; ^6^ School of Medicine University of Nottingham Queens Medical Centre Nottingham UK; ^7^ Faculty of Nursing, Midwifery and Palliative Care King’s College London London UK; ^8^ NIHR Exeter Clinical Research Facility Royal Devon and Exeter NHS Foundation Trust Exeter UK; ^9^ Institute of Biomedical and Clinical Science College of Medicine and Health University of Exeter Exeter UK; ^10^ The Royal Marsden NHS Foundation Trust London UK

**Keywords:** adaptation, barrier, experience, fundamental care, nurses, nursing, support, systematic review

## Abstract

**Aim:**

This systematic review identifies, appraises and synthesizes the evidence on the provision of fundamental nursing care to hospitalized patients with a highly infectious virus and the effectiveness of adaptations to overcome barriers to care.

**Design:**

Systematic review.

**Data Sources:**

In July 2020, we searched Medline, PsycINFO (OvidSP), CINAHL (EBSCOhost), BNI (ProQuest), WHO COVID‐19 Database (https://search.bvsalud.org/) MedRxiv (https://www.medrxiv.org/), bioRxiv (https://www.biorxiv.org/) and also Google Scholar, TRIP database and NICE Evidence, forwards citation searching and reference checking of included papers, from 2016 onwards.

**Review Methods:**

We included quantitative and qualitative research reporting (i) the views, perceptions and experiences of patients who have received fundamental nursing care whilst in hospital with COVID‐19, MERS, SARS, H1N1 or EVD or (ii) the views, perceptions and experiences of professional nurses and non‐professionally registered care workers who have provided that care. We included review articles, commentaries, protocols and guidance documents. One reviewer performed data extraction and quality appraisal and was checked by another person.

**Results:**

Of 3086 references, we included 64 articles; 19 empirical research and 45 review articles, commentaries, protocols and guidance documents spanning five pandemics. Four main themes (and 11 sub‐themes) were identified. Barriers to delivering fundamental care were wearing personal protective equipment, adequate staffing, infection control procedures and emotional challenges of care. These barriers were addressed by multiple adaptations to communication, organization of care, staff support and leadership.

**Conclusion:**

To prepare for continuation of the COVID‐19 pandemic and future pandemics, evaluative studies of adaptations to fundamental healthcare delivery must be prioritized to enable evidence‐based care to be provided in future.

**Impact:**

Our review identifies the barriers nurses experience in providing fundamental care during a pandemic, highlights potential adaptations that address barriers and ensure positive healthcare experiences and draws attention to the need for evaluative research on fundamental care practices during pandemics.

## 1 INTRODUCTION

The emergence of the SARS‐CoV‐2 virus (COVID‐19) has highlighted the importance of the nursing profession and has challenged nursing practice. COVID‐19 has required nurses to adapt their approaches to care due to redeployment (Bagnasco et al., 2020) from their usual environments to care for patients with this highly infectious virus. Many nurses are inexperienced in the strict isolation (Gammon & Hunt, 2018) care procedures required for infection prevention and control (World Health Organization, 2016). The COVID‐19 pandemic has required nurses to implement unfamiliar practices, including but not limited to enhanced infection control procedures (Verbeek et al., 2020).

Commentators on previous pandemics have noted that nurses’ ability to do their jobs was compromised:

The establishment/maintenance of therapeutic nurse‐client relationship required additional time given the barriers of mask, gloves and gowns. p4 (Canadian Nurses Association, 2003).

Restrictions on visitors were difficult for staff because family members are usually involved in the social, psychological and, to some extent, physical care of patients. p6 (Baumann et al., 2003) (Baumann, Blythe, Underwood, & Dzuiba, Capacity, casualization, and continuity: the impact of SARS Toronto, Canada)

Interaction time decreased, and patients began to feel more abandoned. p28 (Registered Nurses Association of Ontario, 2003).

This highlights how nursing care can be a key determinant of patient experience (Graham et al., 2018; Murrells et al., 2013) and satisfaction (Aiken et al., 2017) and is causally linked to safety, clinical effectiveness, care quality and treatment outcomes including mortality and overall service use (Black et al., 2014; Darzi, 2008; Department of Health, 2013; Doyle et al., 2013; Vincent & Amalberti, 2016). It raises concerns that when nursing care is sub‐optimal, patients experience health care negatively (Rathert et al., 2013; Suhonen et al., 2005), leading to wider patient safety failures (Bureau of Health Information, 2014; Department of Health, 2013; Garling, 2008; Kalisch, 2006).

### 1.1 Background

Although many nurses have specialized in discrete areas of practice, all nurses are expected to deliver or supervize others in meeting patients’ ‘fundamental’ care needs. These have been defined as safety, comfort, communication, dignity, respiration, privacy, eating and drinking, respecting choice, elimination, mobility, personal cleansing and dressing, expressing sexuality, temperature control, rest and sleep (Kitson et al., 2010). Activities by nurses to address these areas can be categorized as physical (physical hands on care), relational (establishing a patient/nurse relationship) and psychosocial (wellbeing and mental health) (Feo et al., 2018).

Despite considerable theoretical work (Feo et al., 2018; Kitson et al., 2010), the empirical evidence for specific fundamental care nursing interventions is largely absent. In a review of 149 empirical studies of fundamental nutrition, toileting, mobility and hygiene interventions, all but 13 trials were of low quality and at serious risk of bias. Only one multi‐component intervention which could be used in general nursing practice found effects in favour of the intervention on mobility and incontinence frequency (Richards et al., 2018). This review was not able to provide guidance on which nursing techniques were most effective for delivering fundamental nursing care in these areas.

There have been no reviews specifically for fundamental nursing care in pandemic situations, either concerning the impact of pandemics on nursing or the effectiveness of specific procedures to deliver fundamental care.

Therefore, as part of the intervention development phase (Medical Research Council, 2008) of a trial testing a specific fundamental nursing care protocol for COVID‐19, we undertook a systematic review of the impact of COVID‐19, and other infectious conditions requiring isolation, on the provision of fundamental nursing care and the techniques required by nurses caring for these patients.

## 2 THE REVIEW

### 2.1 Aims

To answer the following research questions:

- What is the impact of COVID‐19 and other conditions requiring isolation on the provision of fundamental nursing care to patients with COVID‐19 in hospital?
- How have adaptations to fundamental nursing care practices as a result of COVID‐19 and other conditions requiring isolation impacted overall patient experience, care quality, functional ability and treatment outcomes for patients with COVID‐19 in hospital?
- What are the areas of fundamental nursing care, for patients with COVID‐19 in hospital, that are evident/missing in published protocols and guidance?

To answer these questions, we will identify, appraise and synthesize the evidence on:

- the impact of COVID‐19 and other pandemic infectious conditions requiring isolation on the provision of fundamental nursing care to patients in hospital;
- the effectiveness of adaptations to overcome these barriers in terms of overall patient experience, care quality, functional ability and treatment outcomes.

We will also present a summary of the available protocols, guidance and research related to specific aspects of fundamental care during a pandemic.

### 2.2 Design

We undertook this systematic review according to best practice guidance (Higgins et al., 2020) and report it according to PRISMA reporting standards (Moher et al., 2009). We registered the protocol with PROSPERO (CRD42020200914).

The patient advisory group for the wider project which included patients with experience of hospitalization due to COVID‐19 was involved in informing all stages of the research and in particular development of the protocol and search strategy as well as the interpretation of the review's findings.

### 2.3 Search methods

A set of key papers was curated from the relevant included studies in two recently published relevant systematic reviews (Pentecost et al., 2020; Richards et al., 2018) and used to develop the search strategy. We used both free text and, where relevant and available, controlled vocabulary terms (e.g. MeSH) and the strategy was peer reviewed and edited by our information specialist (AB) (Example search strategy for Medline in Appendix A).

We searched for studies on 26th July 2020 in the following seven databases: Medline, PsycINFO (OvidSP), CINAHL (EBSCOhost), BNI (ProQuest), WHO COVID‐19 Database (https://search.bvsalud.org/) MedRxiv (https://www.medrxiv.org/) and bioRxiv (https://www.biorxiv.org/) from 2016 onwards along with a search in Google Scholar using Publish or Perish software.

We searched the TRIP database and NICE Evidence for guidance and protocols using the terms ‘pandemic and nursing’ or ‘covid and nursing’. All identified guidelines were full text searched using the term ‘nurse’ to determine relevancy. In response to stakeholder feedback, we undertook additional searches for guidelines in the TRIP and NICE databases using the terms ‘covid and bathing’, ‘covid and nutrition’ and ‘covid and fundamental care’.

In response to stakeholder feedback, we undertook forwards citation searching of a relevant paper (Groven et al., 2017) identified in the database searches and carried out further targeted searching for studies on wipes and washing on 24th August 2020 in the WHO COVID‐19 database, Medline (OvidSP), CINAHL (EBSCOhost) and Nexus.

We carried out forwards citation searching, using Web of Science, as well as reference checking (backwards citation searching) of all included studies. You can observe where each of the included studies were identified in the search summary table (Bethel et al., 2021) in Appendix B.

### 2.4 Eligibility criteria

We included any quantitative or qualitative study reporting (i) the views, perceptions and experiences of patients who have received fundamental nursing care whilst in hospital with COVID‐19, MERS, SARS, H1N1 or Ebola Virus Disease (EVD) and (ii) the views, perceptions and experiences of professional nurses and non‐professionally registered care workers who have provided that care. We were interested in the impact of COVID‐19, MERS, SARS, H1N1 and EVD on the provision of fundamental nursing care and adaptations to fundamental nursing care procedures as a result of the infection. In addition to reports of empirical studies, we also included review articles, commentaries, study reports, case studies, protocols and guidance documents related to COVID‐19. We did this to ensure that COVID‐19 specific information could be incorporated.

We excluded studies where patients were invasively ventilated, as this review was part of the development process for an intervention that does not include this patient group. We also excluded studies that reported experiences of providing and receiving medical care, studies where fundamental nursing care was provided by other registered, health care professionals and studies that were conducted outside of the hospital setting.

Similar to Kitson's model of fundamental nursing care (Kitson et al., 2010), we defined fundamental nursing care as in Table 1.

**TABLE 1:** Fundamental care defined

Physical care	Relational care
Personal cleansing (including oral/mouth care) and dressing	Establishing a relationship with patients
Toileting needs	Talking and listening to patients
Eating and drinking	Non‐verbal communication with patients
Rest and sleep	Shared decision making with patients
Mobility	Dignity and respect needs
Comfort (pain management, breathing easily, temperature control)	Communicating with patients’ relatives, carers and significant others
Safety (risk assessment and management, infection prevention, minimising complications)	Emotional wellbeing and anxiety and low mood
Medication management	Respecting values and beliefs

We did not impose any date or geographical restrictions but only studies published in the English language were included.

### 2.5 Study selection

As an initial calibration exercise, all reviewers (JTC/RA/RW) applied inclusion and exclusion criteria to the same sample (*n* = 100) of search results. After discussion in a group meeting, minor revisions were made to the eligibility criteria to enable more consistent reviewer interpretation and judgement.

We then applied the revised inclusion and exclusion criteria to the title and abstract of each identified citation. Two reviewers (JTC, RA or RW) independently screened 50% of the citations; the remaining citations were screened by just one reviewer (JTC, RA or RW). One reviewer (RA or RW) independently screened all excluded abstracts. All reviewers then piloted inclusion and exclusion criteria on five full text articles (JTC, RA or RW) and decisions were discussed as a group. One reviewer (JTC, RA or RW) screened the full texts of all remaining articles. A second reviewer (RA or RW) independently screened the excluded articles. At all stages, disagreements were resolved through discussion or referral to a third reviewer (JTC, RA or RW) as required.

We used Endnote X8 software to support reference management and the study selection process.

### 2.6 Quality appraisal

One reviewer performed quality appraisal, and this was checked by another person, with consensus achieved through discussion. For empirical quantitative studies, we used the tool developed by the Effective Public Health Practice Project (1998). For qualitative studies, we used the Critical Appraisal Skills Programme checklist for qualitative studies (Critical Appraisal Skills Programme, 2018). For commentary pieces, we used the Joanna Briggs Institute Checklist for Text and Opinion (JBICTO) (McArthur et al., 2015;13(3):188–195, 2015). We did not conduct quality appraisal of the protocols.

### 2.7 Data abstraction

We developed and piloted a standardized data extraction coding set on a selection of included studies and adapted it for use with commentaries. We then used it to collect the following information from each study included at the full text stage: study details (such as author, date of publication, title, study design, topic area, study focus/aim, population involved, ward setting and virus), population details (such as patient/nurse/family, mean age, ethnicity, socioeconomic status, gender and other equity characteristics), fundamental nursing care details, a description of the protocol (as appropriate), type of perception/experience obtained (for qualitative studies only) and the impact discussed or evaluated.

For protocols and guidance documents, we extracted the aspect of fundamental care covered, the guidance provided and the strength of the evidence/guideline (i.e. was it evidence‐based, consensus‐based, expert‐based or opinion‐based guidance).

One reviewer performed data extraction, which was checked by a second, with consensus achieved through discussion with a third person as an arbiter, when required.

### 2.8 Synthesis

We classified data as reporting barriers to the provision of care, adaptations to the provision of care or protocols developed for aspects of fundamental nursing care. We used Microsoft Excel (2013) to present the map of the areas of fundamental care and the type of evidence available.

After initial familiarization with the papers and having extracted the data relevant to our questions, we selected one of the included qualitative research articles, a phenomenological study of healthcare workers in the H1N1 influenza pandemic by Corley et al. (2010), as an index paper for the synthesis, since the themes resonated with the data that we had extracted from the included empirical studies. We used seven of the eight themes proposed by Corley et al. (2010) as a framework to structure coding of the data from the empirical studies which highlighted barriers to the provision of care (the eighth theme was related to a procedure not covered by this review). We then sought to add data from the COVID‐19 pandemic perspective extracted from the commentary pieces, review articles, protocols and guidelines that either supported, or added new information, to that gleaned through research in pandemics. We used four of the seven themes in the framework to reflect four main themes and included the three remaining themes (‘new roles for staff’, ‘staff morale’ and ‘fear and anxiety’) as sub‐themes under those four main themes. We then grouped the rest of the data into sub‐themes relating to specific barriers to the provision of care.

To synthesize the data on adaptations, we reflected on the themes identified in the synthesis of barriers above and cross‐checked whether the adaptations were related to those barriers. We found several key concepts that cut across the identified barriers, in that many of the barriers could be addressed using multiple different adaptations, and that some adaptations addressed more than one barrier. Consequently (and to avoid repetition in the text), the adaptations were collated into new themes and linked to the relevant barriers.

Due to the need to work remotely during the COVID‐19 pandemic, we used Google Jamboard (Google, 2020) to conduct the framework synthesis. One reviewer (JTC, RA or RW) conducted the initial analysis of each paper, which was checked and discussed with a second reviewer.

We collated the protocols identified in the literature into a table to help us map out the areas of fundamental care for which plans for, and potential adaptations of, care of patients during a pandemic either had or had not been developed. We then added the guidance, research literature, reviews and commentaries to this table (gap map) to further enable us to identify areas of fundamental care that are lacking guidance and research to inform pandemic‐specific adaptations to care.

## 3 RESULTS

**FIGURE 1:**
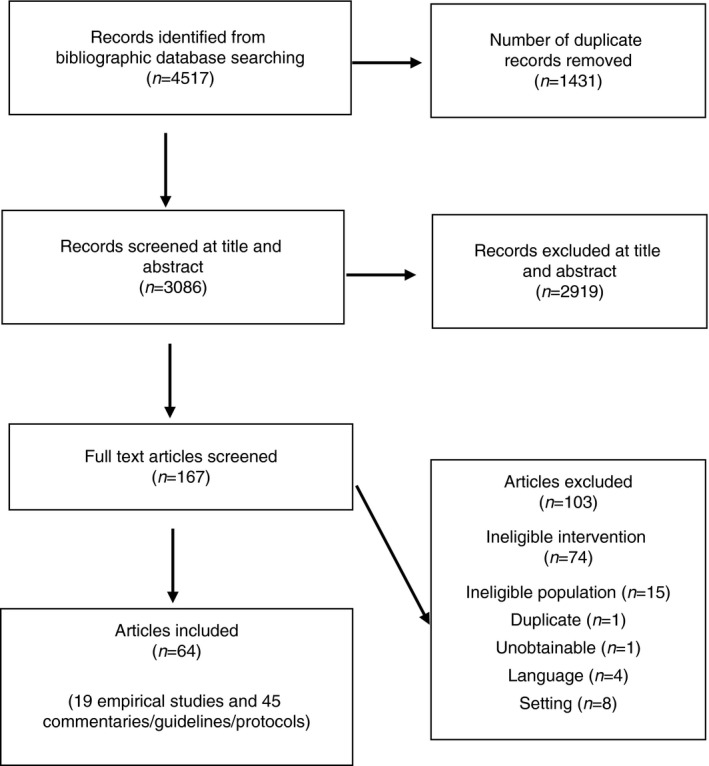
PRISMA flow diagram (in text)

Our systematic search generated 4517 references. We removed 1449 duplicate references, leaving 3086 references to screen at the title and abstract stage. We excluded 2919 references at that stage leaving 167 full texts to screen against the inclusion and exclusion criteria. After full text screening, we included 64 full text references; screening decisions and reasons for exclusion are provided in the PRISMA flow diagram below (Figure 1).

### 3.1 Characteristics of included studies

Of the 64 included papers, 19 were empirical research articles (Table 2) and the remaining 45 were review articles, commentaries, protocols and guidance documents (Table 3). These latter types of information are referred to as ‘commentaries’ below.

**TABLE 2:** Characteristics of included papers of research studies

Author, year	Country	Goal/Aim of paper	Nurse led/involved as author	Key FNC (physical, relational, both)	Aspects of FNC discussed (barriers, solutions, effect on everyday care)	How paper informs FNC
Fundamental care: physical						
Liu, Zhai, et al. (2020) *Qualitative study*	China	To explore the experiences of nurses caring for patients with *COVID‐19*	Yes	Physical	Nurse support Safety (risk assessment and management, infection prevention and minimizing complications)	Experiences/barriers to care
Fundamental care: relational						
Holmgren et al. (2019) *Mixed methods study*	Sweden	To describe nurses’ experiences of health concerns, teamwork, leadership and management and knowledge transfer during an *Ebola* outbreak in West Africa	Yes	Relational	Nurse support	Adapting care
Kuntz et al. (2020) *Mixed methods study*	USA	To describe implementation of telemedicine to facilitate electronic family meetings to facilitate in‐patient palliative care for *COVID‐19* patients	No	Relational	Communicating with patients’ relatives, carers and significant others Establishing a relationship with patients Respecting values and beliefs	Adapting care Experiences/barriers to care
Umoren et al. (2020) *Observational study*	USA	To describe the use of telemedicine during ward rounds for patients with *COVID‐19* in isolation wards	Yes	Relational	Organization of care Safety (risk assessment and management, infection prevention and minimizing complications)	Adapting care Experiences/barriers to care
Viswanathan et al. (2020) *Observational study*	USA	To describe the use of a virtual group‐based support intervention for nurses during the *COVID‐19* pandemic	Yes	Relational	Talking and listening to patients Nurse support	Experiences/barriers to care Adapting care
Fundamental care: both						
Andertun et al. (2017) *Qualitative study*	Sweden	To describe the experience of caring for patients with *Ebola*	Yes	Both	Safety (risk assessment and management, infection prevention and minimizing complications) Talking and listening to patients Organization of care	Experiences/barriers to care Adapting care
Chan, Chung, et al. (2006) *Observational study*	China	Exploring modular vs conventional models of nursing care in the management of *SARS* patients	Yes	Both	Physical care Organization of care Establishing a relationship with patients	Experiences/barriers to care Adapting care
Chan, Leung, et al. (2006) *Observational study*	China	Nursing issues for caring for children with suspected *SARS* and proposition of a family‐centred model of care	Yes	Both	Communicating with patients’ relatives, carers and significant others Safety (risk assessment and management, infection prevention and minimizing complications) Nurse support Emotional wellbeing, anxiety and low mood	Experiences/barriers to care Adapting care Guidance/Protocol/Recommendations
Chan et al. (2008) *Trial/Cohort/Case–Control*	China	To compare a modular vs conventional model of nursing care in the management of *SARS* patients	Yes	Both	Organization of care Physical care Toileting Communicating with patients’ relatives, carers and significant others Nurse support Establishing a relationship with patients	Experiences/barriers to care Adapting care
Cheng et al. (2005) *Qualitative study*	China	Retrospective exploration of healthcare workers verbal and behavioural response to patients with *SARS*	Unsure	Both	Talking and listening to patients Emotional wellbeing, anxiety and low mood Physical care Toileting	Experiences/barriers to care
Corley et al. (2010) *Qualitative study*	Australia	To describe the lived experience of nurses and medical staff caring for patients with *H1N1*	Yes	Both	Safety (risk assessment and management, infection prevention and minimizing complications) Dignity and respect Physical care Nurse support	Experiences/barriers to care Adapting care
Kang et al. (2018) *Qualitative study*	South Korea	To explore working experiences of nurses during *MERS* outbreak	Yes	Both	Establishing a relationship with patients Safety (risk assessment and management, infection prevention and minimizing complications) Physical care Nurse support Organization of care	Experiences/barriers to care Adapting care
Kim, 2018 *Qualitative study*	South Korea	To explore nurses’ experiences of care for patients with *MERS*	Yes	Both	Communicating with patients’ relatives, carers and significant others Establishing a relationship with patients Safety (risk assessment and management, infection prevention and minimizing complications)	Experiences/barriers to care
Lee et al. (2020) *Qualitative study*	South Korea	To explore the experience of nurses caring for patients with *MERS*	Yes	Both	Nurse support Safety (risk assessment and management, infection prevention and minimizing complications)	Experiences/barriers to care
Liu and Liehr (2009)	China	To explore the experiences of nurses caring for patients with *SARS*	Yes	Both	Nurse support Safety (risk assessment and management, infection prevention and minimizing complications)	Experiences/barriers to care Adapting care
Liu, Luo, et al. (2020) *Qualitative study*	China	To describe the experiences of these health‐care providers in the early stages of the outbreak. (*COVID‐19*)	Yes	Both	Eating and drinking Toileting needs Talking and listening to patients Comfort Physical care	Experiences/barriers to care
Shih et al. (2007) *Qualitative study*	Taiwan	To identify the stage‐specific difficulties encountered by Taiwan's frontline nurses during the *SARS* outbreak	Yes	Both	Organization of care Nurse support Safety (risk assessment and management, infection prevention and minimizing complications)	Experiences/barriers to care Adapting care
Shih et al. (2009) *Qualitative study*	Taiwan	To explore Taiwan's nurse leaders’ reflections and experiences of the difficulties they encountered and survival strategies they employed during the *SARS* outbreak	Yes	Both	Nurse support Safety (risk assessment and management, infection prevention and minimizing complications) Communicating with patients’ relatives, carers and significant others	Experiences/barriers to care Adapting care Guidance/Protocol/Recommendations
Tiwari et al. (2003) *Qualitative study*	Hong Kong	Provide a descriptive analysis of nursing care during the *SARS* outbreak	Yes	Both	Safety (risk assessment and management, infection prevention and minimizing complications) Emotional wellbeing, anxiety and low mood Nurse support Organization of care	Experiences/barriers to care Patients experience of care Adapting care

Abbreviation: FNC, fundamental care

Of the 19 research articles, one concerned the physical aspects of fundamental care and four the relational aspect, with 14 articles about both. Studies were conducted in China (*n* = 7), USA (*n* = 3), South Korea (*n* = 3), Sweden (*n* = 2), Taiwan (*n* = 2), Hong Kong (*n* = 1) and Australia (*n* = 1) and involved caring for patients with COVID‐19 (*n* = 5), SARS (*n* = 8), MERS (*n* = 3), EVD (*n* = 2) and H1N1 (*n* = 1). Twelve studies used qualitative methods (Andertun et al., 2017; Cheng et al., 2005; Corley et al., 2010; Kang et al., 2018; Kim, 2018; Lee et al., 2020; Liu & Liehr, 2009; Liu, Luo, et al., 2020; Liu, Zhai, et al., 2020; Shih et al., 2007; Shih et al., 2009; Tiwari et al., 2003); researchers used individual interviews (*n* = 7), a mix of focus groups and interviews (*n* = 4) and focus groups only (*n* = 1). Seven studies used quantitative methods (45–51); four non‐experimental (two case studies, one retrospective case series and one simulation study) (Chan, Chung, et al., 2006; Chan, Leung, et al., 2006; Umoren et al., 2020; Viswanathan et al., 2020), one had a pre–post design (Chan et al., 2008) and two used surveys (Holmgren et al., 2019; Kuntz et al., 2020). The majority of studies (17/19) were nurse led or included a nurse in the research team. Eight of the qualitative studies collected data from nurses (*n* = 8), two from nurses and medical practitioners and two from patients/survivors. Sample sizes ranged from six to 200 participants.

The remaining 45 articles were commentaries (*n* = 30), protocols (*n* = 8), reviews (*n* = 4) or guidance (*n* = 3). Eleven addressed the physical aspects of fundamental care (Aguila et al., 2020; Anderson, 2020a, 2020b; Caccialanza, 2020; Cena et al., 2020; Chapple et al., 2020; Cintoni et al., 2020; DeCastro et al., 2020; Dingfield et al., 2020; Sharma et al., 2020; Wang, Sun, et al., 2020), 19 the relational aspect (Adams, 2020; Bagnasco et al., 2020; Bouchoucha & Bloomer, 2020; Brown‐Johnson et al., 2020; Cathcart, 2020; Chochinov et al., 2020; Diamond et al., 2020; Fang et al., 2020; Fausto et al., 2020; Hart et al., 2020; Hofmeyer et al., 2020a, 2020b; Humphreys et al., 2020; Maben et al., 2020; Morley et al., 2020; Neville, 2020; Taylor, 2020; Wakam et al., 2020) and 17 both aspects (Buheji & Buhaid, 2020; Danielis & Mattiussi, 2020; deLima Thomas et al., 2020; Estella, 2020; Fan et al., 2020; Fedele, 2020; Feder et al., 2020; Maltby & Conroy, 2020; Martland & Huffines, 2020; Newby et al., 2020; Pahuja & Wojcikewych, 2020; Pettis, 2020; Rangachari & L. Woods, 2020; Rosa et al., 2020; Tsai et al., 2020; Wang, Zeng, et al., 2020). First authors of the papers were based in the USA (*n* = 23), UK (*n* = 5), Italy (*n* = 5), Australia (*n* = 3), Taiwan (*n* = 2), Canada (*n* = 1), China (*n* = 1), India (*n* = 1), the Philippines (*n* = 1), Bahrain (*n* = 1), Spain (*n* = 1) and Singapore (*n* = 1). All 45 articles addressed COVID‐19.

Study quality ratings are shown in Tables 4 and 5. All seven quantitative studies were rated as weak according to the EPHPP global rating (Effective Public Health Practice Project, 1998), due to study design, lack of accounting for confounders and poor reporting of withdrawals from the studies and analysis. The 12 studies that used qualitative methods were largely well conducted with most studies reporting no more than one or two ‘weak’ elements other than one study (Kuntz et al., 2020) which had six ‘weak’ elements.

**TABLE 3:** Characteristics of included commentaries

Author, year	Country	Goal/Aim of paper	Nurse led/involved as author	Key FNC (physical, relational, both)	Aspects of FNC discussed (barriers, solutions and effects on everyday care)	How paper informs FNC
Fundamental care: physical						
Aguilla et al. (2020) *Protocol*	Philippines	To review relevant data on nutrition and gastroenterology in relation to *COVID‐19*	Unsure	Physical	Eating and drinking	Guidance/protocol/recommendations
Anderson (2020a) *Literature Review*	UK	To summarize nutrition guidance for patients with *COVID‐19* for nurses	Yes	Physical	Eating and drinking	Guidance/protocol/recommendations
Anderson (2020b) *Literature Review*	UK	To summarize nutrition guidance for patients with *COVID‐19* for nurses	Yes	Physical	Eating and drinking Personal cleansing (including oral/mouth care) and dressing	Guidance/protocol/recommendations
Caccialanza (2020) *Protocol*	Italy	Protocol for early nutritional supplementation of non‐critically ill patients with *COVID ‐19*.	Yes	Physical	Eating and drinking	Guidance/protocol/recommendations
Cena et al. (2020) *Protocol*	Italy	Position paper on the nutritional management of patients with *COVID‐19* disease	No	Physical	Eating and drinking	Guidance/protocol/recommendations
Chapple et al. (2020) *Protocol*	Australia	Evidence‐based nutrition management for critically ill and acutely unwell hospitalized patients during *COVID‐19*	Yes	Physical	Eating and drinking	Guidance/protocol/recommendations
Cintoni et al. (2020) *Opinion/Editorial*	Italy	Presentation of the nutritional strategy for patients with *COVID‐19*	Unsure	Physical	Eating and drinking	Adapting care Guidance/protocol/recommendations
DeCastro et al. (2020) *Opinion/Editorial*	USA	To use a checklist to standardize inter‐professional care and communication across multiple non‐ICU *COVID‐19* Units	Yes	Physical	Organization of care	Adapting care
Dingfield et al. (2020) Opinion/Editorial	USA	To adapt tools to guide pain, dyspnea and anxiety management at end of life for patients with *COVID‐19*	Yes	Physical	Medication management	Adapting care Guidance/protocol/recommendations
Sharma et al. (2020) *Literature review*	India	To describe the role of nurses in the management of patients with *COVID‐19*	Yes	Physical	Personal cleansing (including oral/mouth care) and dressing Rest and sleep	Guidance/protocol/recommendations
Wang, Zeng, et al. (2020) and Wang, Sun, et al. (2020) *Consensus statement*	Taiwan	Provide guidance for clinical practice in *COVID‐19*	Yes	Physical	Comfort (pain management, breathing easily and temperature control) Safety (risk assessment and management, infection prevention and minimizing complications)	Guidance/protocol/recommendations
Fundamental care: relational						
Adams (2020) *Opinion/Editorial*	USA	To describe challenges and recommendations around palliative care for patients with *COVID‐19*	No	Relational	Communicating with patients’ relatives, carers and significant others; Establishing a relationship with patients Nurse support	Experiences/barriers to care Adapting care
Bagnasco et al. (2020) *Opinion/Editorial*	Italy	To share the experience of caring for patients with *COVID‐19* and lessons learned	Yes	Relational	Establishing a relationship with patients Communicating with patients’ relatives, carers and significant others Organization of care Nurse support	Experiences/barriers to care Adapting care
Bouchoucha and Bloomer (2020) *Opinion/Editorial*	Australia	To highlight the importance of maintaining family‐centred care for end‐of‐life care in times of visitor restrictions (*COVID‐19*)	Yes	Relational	Communicating with patients’ relatives, carers and significant others Shared decision making with patients	Adapting care
Brown‐Johnson et al. (2020) *Opinion/Editorial*	USA	To share experience of PPE portraits a way of humanizing PPE (*COVID‐19*)	Yes	Relational	Non‐verbal communication with patients Talking and listening to patients	Adapting care
Cathcart (2020) *Opinion/Editorial*	USA	To offer new nurse managers basic tenets of leadership during *COVID‐19*	Yes	Relational	Dignity and respect needs Nurse support Organization of care	Adapting care Guidance/protocol/recommendations
Chochinov et al. (2020) *Opinion/Editorial*	Canada	Perspective on barriers to dying with dignity during *COVID‐19*	No	Relational	Dignity and respect needs Establishing a relationship with patients	Experiences/barriers to care Adapting care
Diamond et al. (2020) *Opinion/Editorial*	USA	To highlight equitable care to patients with limited dominant language proficiency amid the *COVID‐19* pandemic	Unsure	Relational	Talking and listening to patients	Experiences/barriers to care Adapting care
Fang et al. (2020) *Opinion/Editorial*	USA	Hospital telehealth communication solutions for patients, family and staff during isolation (*COVID_19*)	No	Relational	Establishing a relationship with patients Communicating with patients’ relatives, carers and significant others	Adapting care
Fausto et al. (2020) *Protocol*	USA	Development of a palliative care strategy in response to the *COVID‐19* pandemic	Unsure	Relational	Shared decision making Organization of care	Adapting care Guidance/protocol/recommendations
Hart et al. (2020) *Opinion/Editorial*	USA	To provide a framework for family‐centred care (*COVID‐19*)	No	Relational	Dignity and respect Communicating with patients’ relatives, carers and significant others Respecting values and beliefs	Experiences/barriers to care Adapting care
Hofmeyer et al. (2020a) *Opinion/Editorial*	UK	How health system leaders can implement relevant organizational interventions to reduce caregiver burnout and promote engagement and compassionate practice during the *COVID‐19* pandemic	Yes	Relational	Nurse support Dignity and respect	Experiences/barriers to care Adapting care
Hofmeyer et al. (2020b) *Opinion/Editorial*	UK	To explain how nurses, midwives and students can better care for themselves so they can better care for others during the *COVID‐19* pandemic	Yes	Relational	Nurse support Communicating with patients’ relatives, carers and significant others	Adapting care
Humphreys et al. (2020) *Opinion/Editorial*	USA	To describe rapid and ongoing implementation of tele‐palliative medicine consultation for patients with *COVID‐19*	Yes	Relational	Establishing a relationship with patients Communicating with patients’ relatives, carers and significant others Shared decision making Non‐verbal communication	Experiences/barriers to care Adapting care
Maben et al. (2020) *Protocol*	UK	To provide suggestions to support nurses at work during *COVID 19*	Yes	Relational	Nurse support Organization of care	Guidance/protocol/recommendations
Morley et al. (2020) *Consensus Statement*	USA	Discuss five types of moral distress and provide suggestions for responding to moral distress in staff during *COVID 19*	Yes	Relational	Nurse support	Adapting care Guidance/protocol/recommendations
Neville (2020) *Opinion/Editorial*	USA	Going above and beyond for dying patients and their families in *COVID‐19*	No	Relational	Dignity and respect needs Establishing a relationship with patients Communicating with patients’ relatives, carers and significant others Emotional wellbeing, anxiety and low mood Organization of care	Experiences/barriers to care Adapting care
Taylor, (2020) *Opinion/Editorial*	USA	The pros and cons of nurses offering to pray with patients with *COVID‐19*	Yes	Relational	Respecting values and beliefs	Adapting care
Wakam et al. (2020) *Opinion/Editorial*	USA	Perspective on how compassionate care in patients with *COVID‐19* can be done better	No	Relational	Establishing a relationship with patients Communicating with patients’ relatives, carers and significant others	Adapting care
Fundamental care: both						
Buheji and Buhaid (2020) *Protocol*	Bahrain	To propose a nurse‐specific human factor framework for *COVID‐19* care	Yes	Both	Organization of care Safety (risk assessment and management, infection prevention and minimizing complications) Nurse support	Adapting care
Danielis and Mattiussi (2020) *Opinion/Editorial*	Italy	Perspective on the aspects of fundamental care that have been lost in caring for *COVID‐19* patients	Yes	Both	Physical care Communicating with patients’ relatives, carers and significant others Emotional wellbeing, anxiety and low mood Talking and listening to patients Dignity and respect Safety (risk assessment and management, infection prevention and minimizing complications)	Experiences/barriers to care
deLima Thomas et al. (2020) *Protocol*	USA	Consolidated palliative care educational materials to serve as a resource to non‐palliative care clinicians during *COVID‐19*	Yes	Both	Physical care Communicating with patients’ relatives, carers and significant others Talking and listening to patients Comfort Organization of care	Adapting care Guidance/protocol/recommendations
Estella (2020) *Opinion/Editorial*	Spain	To promote a series of measures aimed at improving communication between critically ill patients with *COVID‐19* in isolation and their family members	No	Both	Communicating with patients’ relatives, carers and significant others Dignity and respect needs Emotional wellbeing and anxiety and low mood Shared decision making Establishing a relationship with patients Comfort	Experiences/barriers to care Adapting care
Fan et al. (2020) *Opinion/Editorial*	Singapore	To highlight the concerns of patients in isolation care units: learnings from *COVID‐19*	Yes	Both	Communicating with patients’ relatives, carers and significant others Emotional wellbeing and anxiety and low mood Eating and drinking Non‐verbal communication	Experiences/barriers to care Adapting care
Fedele (2020) *Opinion/Editorial*	Australia	To reflect on the experience of nursing patients with *COVID‐19* in an ICU	Yes	Both	Safety (risk assessment and management, infection prevention and minimizing complications) Organization of care	Experiences/barriers to care
Feder et al. (2020) *Opinion/Editorial*	USA	Palliative care strategies for clinicians to give comfort for patients with *COVID‐19* and families	Yes	Both	Physical care Communicating with patients’ relatives, carers and significant others Dignity and respect Talking and listening to patients Respecting values and beliefs Emotional wellbeing, anxiety and low mood	Experiences/barriers to care Adapting care
Maltby and Conroy (2020) *Opinion/Editorial*	USA	To provide guidance around *COVID‐19* to nurse leaders in response to results from a statewide nurse survey	Yes	Both	Safety (risk assessment and management, infection prevention and minimizing complications) Nurse support	Guidance/protocol/recommendations
Martland and Huffines (2020) *Opinion/Editorial*	USA	Nursing leadership and administrative considerations for fostering nurse resilience in pandemics (*COVID‐19*)	No	Both	Organization of care Nurse support Safety (risk assessment and management, infection prevention and minimizing complications) Physical care	Adapting care
Newby et al. (2020) *Opinion/Editorial*	USA	To share tips, tricks, modifications and techniques found to be useful during the *COVID‐19* pandemic and to facilitate the nursing communication	Yes	Both	Organization of care Safety (risk assessment and management, infection prevention and minimizing complications)	Experiences/barriers to care Adapting care
Wang et al. (2020) *Consensus Statement*	China	Development of a holistic care consensus statement for patients with *COVID 19*	Yes	Both	Physical Emotional wellbeing, anxiety and low mood	Guidance/protocol/recommendations
Pahuja and Wojcikewych (2020) *Opinion/Editorial*	USA	A case study demonstrating the challenges to end‐of‐life care for patients with *COVID‐19*	Yes	Both	Communicating with patients’ relatives, carers and significant others Establishing a relationship with patients Respecting values and beliefs Medication management	Experiences/barriers to care Adapting care
Pettis (2020) *Opinion/Editorial*	USA	To highlight the value of gerontological‐trained specialist nurses for patient care in pandemic times (*COVID‐19*)	Yes	Both	Organization of care Safety (risk assessment and management, infection prevention and minimizing complications) Non‐verbal communication Shared decision making with patients	Adapting care
Rangachari and L. Woods (2020) *Opinion/Editorial*	USA	To discuss the issues for healthcare organizations in relation to organizational resilience, staff retention and patient safety (*COVID‐19*)	No	Both	Safety (risk assessment and management, infection prevention and minimizing complications) Organization of care Nurse support	Adapting care
Rosa et al. (2020) *Literature review*	USA	Palliative care engagement in critical care (*COVID‐19*)	Yes	Both	Comfort (pain management, breathing easily and temperature control) Shared decision making with patients Establishing a relationship with patients Communicating with patients’ relatives, carers and significant others Safety (risk assessment and management, infection prevention and minimizing complications)	Experiences/barriers to care Guidance/protocol/recommendations
Tsai et al. (2020) *Opinion/Editorial*	Taiwan	To highlight some of the ways technology can be used to provide patient care whilst keeping staff safe in *COVID‐19*	Yes	Both	Organization of care Nurse support Safety (risk assessment and management, infection prevention and minimizing complications) Medication Emotional wellbeing, anxiety and low mood	Experiences/barriers to care Adapting care

**TABLE 4:** Quality appraisal for quantitative studies (EPHPP)

Author, year	Selection bias	Study design	Confounders	Blinding	Data collection methods	Withdrawals/dropouts	Analysis	Global rating
Fundamental Care: physical								
Fundamental care: relational								
Holmgren et al. (2019) *Mixed methods study*	Weak	Weak	Weak	Na	Weak	Weak	Weak	Weak
Kuntz et al. (2020) *Mixed methods study*	Moderate	Weak	Weak	Weak	Weak	Moderate	Weak	Weak
Umoren et al. (2020 )*Observational study*	Moderate	Weak	Weak	Na	Moderate	Weak	Weak	Weak
Viswanathan et al. (2020 **)** *Observational study*	Moderate	Weak	Weak	Na	Weak	Weak	Weak	Weak
Fundamental care: both								
Chan, Chung, et al. (2006) *Observational study*	Moderate	Weak	Weak	NA	Moderate	Weak	Moderate	Weak
Chan, Leung, et al. (2006) *Observational study*	Weak	Weak	Weak	NA	Weak	Weak	Weak	Weak
Chan (2008) *Trial/Cohort/Case‐Control*	Moderate	Weak	Moderate	NA	Moderate	Strong	Moderate	Weak

Quality appraisal of the non‐research papers is shown in Table 6. Only the three consensus statements (Morley et al., 2020; Wang, Zeng, et al., 2020; Wang, Sun, et al., 2020) could be graded positively against all elements of the JBICTO (McArthur et al., 2015) with the remaining commentaries generally failing the element that suggests that it is supported by peers, as this was difficult to ascertain.

**TABLE 5:** Quality appraisal for qualitative studies (CASP)

Author, Year	Clear question	Appropriate methods	Appropriate research design	Appropriate recruitment strategy	Appropriate data collection	Researcher role considered	Ethical issues considered	Analysis sufficiently rigorous	Clear statement of findings
Fundamental care: physical									
Liu, Zhai, et al. (2020) *Qualitative study*	Yes	Yes	Yes	Can't tell	Yes	No	Yes	Yes	Yes
Fundamental care: relational									
Holmgren et al. (2019) *Mixed methods study*	Yes	No	Unclear	Yes	Yes	No	Yes	Can't tell	Yes
Kuntz et al. (2020) *Mixed methods study*	Yes	Yes	No	Yes	No	No	No	No	No
Fundamental care: both									
Andertun et al. (2017) *Qualitative study*	Yes	Yes	Yes	Yes	Yes	No	Yes	Yes	Yes
Cheng et al. (2005) *Qualitative study*	Yes	Yes	Yes	Yes	Yes	No	Can't tell	Yes	Yes
Corley et al. (2010) *Qualitative study*	Yes	Yes	Yes	Yes	Yes	No	Yes	Can't tell	Yes
Kang et al. (2018) *Qualitative study*	Yes	Yes	Yes	Yes	Yes	Can't tell	Can't tell	Yes	Yes
Kim (2018) *Qualitative study*	Yes	Yes	Yes	Yes	Yes	Can't tell	Can't tell	Yes	Yes
Lee et al. (2020) *Qualitative study*	Yes	Yes	Yes	Yes	Yes	Yes	Yes	Yes	Yes
Liu and Liehr (2009) *Qualitative study*	Yes	Yes	Yes	Can't tell	Yes	No	Can't tell	Yes	Yes
Liu, Luo, et al. (2020) *Qualitative study*	Yes	Yes	Yes	Can't tell	Yes	No	Yes	Yes	Yes
Shih et al. (2007) *Qualitative study*	Yes	Yes	Yes	Yes	Yes	Can't tell	Can't tell	Yes	Yes
Shih et al. (2009) *Qualitative study*	Yes	Yes	Yes	Yes	Yes	Can't tell	Can't tell	Yes	Yes
Tiwari et al. (2003) *Qualitative study*	Yes	Yes	Yes	Yes	Yes	Can't tell	Can't tell	No	Yes

**TABLE 6:** Quality appraisal for opinion pieces (JBI)

Author, Year	Source of opinion identified	Source has standing/expertise	Focus primarily on aspect of FNC	Is the basis of opinion clearly argued	Reference to extant literature	Opinion supported by peers
Fundamental care: physical						
Anderson (2020a) *Literature Review*	Yes	Yes	Yes	Yes	Yes	Can’t tell
Anderson (2020b) *Literature Review*	Yes	Yes	Yes	Yes	Yes	Can’t tell
Cintoni et al. (2020) *Opinion/Editorial*	Yes	Yes	Yes	Yes	Yes	Can’t tell
DeCastro et al. (2020) *Opinion/Editorial*	Yes	No	Yes	No	No	Can’t tell
Dingfield et al. (2020) Opinion/Editorial	Yes	Yes	No	No	No	Yes
Sharma et al. (2020) *Literature review*	Yes	Unsure	Yes	Yes	Yes	Unsure
Wang, Sun, et al. (2020) *Consensus statement*	Yes	Yes	Yes	Yes	Yes	Yes
Fundamental care: relational						
Adams (2020) *Opinion/Editorial*	Yes	Yes	Yes	Yes	Yes	Can’t tell
Bagnasco et al. (2020) *Opinion/Editorial*	Yes	Yes	No	Yes	No	Can’t tell
Beck (2004) *Opinion/Editorial*	Yes	Yes	Yes	Yes	Yes	Yes
Bouchoucha and Bloomer (2020) *Opinion/Editorial*	Yes	Yes	Yes	Yes	Yes	Can’t tell
Brown‐Johnson et al. (2020) *Opinion/Editorial*	Yes	Yes	Yes	Yes	Yes	Yes
Cathcart (2020) *Opinion/Editorial*	Yes	Yes	Yes	Yes	No	Can’t tell
Chochinov et al. (2020) *Opinion/Editorial*	Yes	Yes	Yes	No	Yes	Can’t tell
Diamond et al. (2020) *Opinion/Editorial*	Yes	Yes	Yes	Yes	Yes	Can’t tell
Fang et al. (2020) *Opinion/Editorial*	Yes	Can’t tell	Yes	Yes	Yes	Can’t tell
Hart et al. (2020) *Opinion/Editorial*	No	Unsure	Yes	No	Yes	Unsure
Hofmeyer (2020a) *Opinion/Editorial*	Yes	Yes	Yes	Yes	Yes	Unsure
Hofmeyer (2020b) *Opinion/Editorial*	Yes	Yes	Yes	Yes	Yes	Unsure
Humphreys et al. (2020) *Opinion/Editorial*	Yes	Yes	No	Yes	Yes	Yes
Morley et al. (2020) *Consensus Statement*	Yes	Yes	Yes	Yes	Yes	Yes
Neville (2020) *Opinion/Editorial*	Yes	Yes	Yes	Yes	No	Can’t tell
Taylor (2020) *Opinion/Editorial*	Yes	Yes	Yes	Yes	Yes	Can’t tell
Wakam et al. (2020) *Opinion/Editorial*	Yes	Yes	Yes	Yes	No	Can’t tell
Fundamental care: both						
Buheji and Buhaid (2020) *Protocol*	Yes	Unsure	Yes	No	Yes	Can’t tell
Danielis and Mattiussi (2020) *Opinion/Editorial*	Yes	Unsure	Yes	No	Yes	Can’t tell
Estella (2020) *Opinion/Editorial*	Yes	Yes	Yes	Yes	Yes	Yes
Fan et al. (2020) *Opinion/Editorial*	Yes	Yes	Yes	Yes	Yes	Can’t tell
Fedele (2020) *Opinion/Editorial*	Yes	Yes	Yes	Yes	No	Unsure
Feder et al. (2020) *Opinion/Editorial*	No	Unsure	Yes	Yes	Yes	Can’t tell
Martland and Huffines (2020) *Opinion/Editorial*	Yes	Yes	No	Yes	Yes	Can’t tell
Newby et al. (2020) *Opinion/Editorial*	Yes	Yes	Yes	Yes	Yes	Can’t tell
Wang et al. (2020) *Consensus Statement*	Yes	Yes	Yes	Yes	Yes	Yes
Pahuja and Wojcikewych (2020) *Opinion/Editorial*	Yes	Yes	Yes	Yes	Yes	Can’t tell
Pettis (2020) *Opinion/Editorial*	Yes	Yes	No	Yes	No	Yes
Rangachari and L. Woods (2020) *Opinion/Editorial*	Yes	Yes	Yes	Yes	Yes	Can’t tell
Rosa et al. (2020) *Literature review*	Yes	Unsure	Yes	Yes	Yes	Unsure
Tsai et al. (2020) *Opinion/Editorial*	Yes	Yes	Yes	Yes	Yes	Can’t tell
Wilson (2015) *Opinion/Editorial*	Yes	Yes	No	Yes	Yes	Can’t tell

We synthesized the available literature to attempt to answer our three research questions. These questions are presented below along with the appropriate findings and discussion.

### 3.2 Question 1: what is the impact of COVID‐19 and other conditions requiring isolation on the provision of fundamental nursing care to patients with COVID‐19 in hospital?

To answer this question we identified, appraised and synthesized the available evidence on:

- the impact of COVID‐19 and other pandemic infectious conditions requiring isolation on the provision of fundamental nursing care to patients in hospital;
- the effectiveness of adaptations to overcome these barriers in terms of overall patient experience, care quality, functional ability and treatment outcomes.

We derived four main themes and 11 sub‐themes from the data describing barriers to the provision of care. Table 7 provides a summary of these themes which are then presented in detail below.

**TABLE 7:** Summary of themes identified from barriers to the provision of fundamental care at the hospital bedside

Theme	Sub theme (barrier)
Impact of PPE	On communication
	On nursing tasks
	On physical health
Adequate staffing	Workload
	New roles for staff
Infection control procedures	Controlling contamination
	Visitors
Emotional challenges of care	Fear and anxiety
	Interaction between patients, staff and family members
	Lack of staff support in work and community
	Burden

#### 3.2.1 Theme 1: personal protective equipment

In this theme, we identified three sub‐themes of the impact that wearing Personal protective equipment (PPE) can have on: communication; the ability to carry out nursing tasks and the physical health of staff.

##### Impact on communication

Wearing PPE interfered with body language and non‐verbal communication, making it difficult for healthcare workers to hear and speak (Andertun et al., 2017; Corley et al., 2010; Umoren et al., 2020; Viswanathan et al., 2020). Commentaries on the impact of COVID‐19 supported these observations (Brown‐Johnson et al., 2020; Danielis & Mattiussi, 2020), highlighting how the loss of non‐verbal communication due to wearing PPE can affect the ‘human connection’ (Brown‐Johnson et al., 2020).

##### Impact on nursing tasks

Wearing PPE, often in multi‐layers, affected healthcare workers dexterity and vision, affecting everyday tasks, such as feeling for veins, position changing and aspirating patients (Lee et al., 2020; Liu & Liehr, 2009; Liu, Luo, et al., 2020). PPE also restricted the amount of time healthcare staff could be with patients due to the amount of time it took to put on and off (Andertun et al., 2017; Viswanathan et al., 2020). This was supported by commentary evidence from nurses working with COVID‐19 (Tsai et al., 2020). Healthcare workers also reported concerns about sufficient provision of PPE and having the appropriate knowledge/training to use it (Corley et al., 2010; Kang et al., 2018; Lee et al., 2020; Liu, Zhai, et al., 2020; Shih et al., 2007) and how this impacted on their confidence in delivering appropriate care safely.

##### Impact on physical health

Several studies highlighted the physical discomfort of PPE, which both directly and indirectly affected the ability to care for patients (Corley et al., 2010; Kang et al., 2018; Lee et al., 2020; Viswanathan et al., 2020). Wearing of PPE was reported to result in headaches, nausea, dermatitis, dehydration and exhaustion—either directly or by the act of not eating and drinking during shifts due to the time it takes to don and doff (Kang et al., 2018; Kim, 2018; Liu, Luo, et al., 2020; Liu, Zhai, et al., 2020). This was also supported by commentary evidence (Fedele, 2020).

#### 3.2.2 Theme 2: adequate staffing

In this theme, we identified two sub‐themes: the impact that inadequate staffing levels have on workload and the need for staff to take on new roles.

##### Workload

Several studies reported increased workload as a result of inadequate staffing levels (Chan et al., 2008; Corley et al., 2010; Liu, Luo, et al., 2020). In some cases, this led to feelings of fatigue, of staff being stretched to their limit, that units could not cope without staff working overtime and that the quality of care was in jeopardy (Chan, Leung, et al., 2006; Corley et al., 2010; Liu & Liehr, 2009). Staff reported eroding of meal breaks—regarded as a very important way of coping with the difficult situation (Corley et al., 2010)—by the time taken to don and doff PPE and also by the activity of the unit. Difficulties were also reported in achieving the correct skill mix to deliver fundamental nursing care to patients. Whilst, increased ancillary staff levels were necessary to cope with additional cleaning, waste generated by PPE, patient transfers and support required when moving critically ill patients, matching the nursing skill mix to the high acuity level of patients, and being able to provide support to less experienced members of staff were also seen as challenges (Corley et al., 2010). These concerns were highlighted in commentaries related to COVID‐19 where exhaustion was reported due to heavy workloads and protective gear (Chochinov et al., 2020).

##### New roles for staff

Several qualitative studies (Corley et al., 2010; Liu, Luo, et al., 2020; Liu, Zhai, et al., 2020) highlighted the challenges for junior staff, retired staff recalled into work and staff familiar only with working in other specialties, in having to cope with patients with complex care needs and unfamiliar environments. Commentaries on the impact of COVID‐19 supported these reports (Bagnasco et al., 2020; Cathcart, 2020). Nurses in the qualitative studies reported the need for more knowledge about infectious diseases, how to care for the psychological needs of patients arising from isolation (Liu & Liehr, 2009) and the need for clear delineation around roles and responsibilities (Chan, Chung, et al., 2006). Although there were issues for staff related to inexperience, there were studies, which highlighted how some nurses relished the challenge of learning new skills and felt a strong responsibility, as a nurse, in having to do so (Kim, 2018). Often new skills had to be learnt and mastered in a very short time (Liu, Luo, et al., 2020), in particular, around technology facilitating communication between staff, patients and families (Kuntz et al., 2020). Other challenges linked to changing roles were leadership (Holmgren et al., 2019), difficulties communicating across different departments and specialities (Liu, Luo, et al., 2020) and frequently changing guidelines.

#### 3.2.3 Theme 3: infection control procedures

In this theme, we identified two subthemes: controlling contamination; impact on visitors.

##### Controlling contamination

Several studies reported that increased levels of vigilance when monitoring patients, the need to ensure visitors wear the correct PPE, lack of or conflicting information about whether to treat patients as infectious, and the need to keep up with frequently changing guidelines added to the workload (Corley et al., 2010; Kang et al., 2018) and contributed to nurses’ stress (Kang et al., 2018). Authors of studies reported that staff found combining infection control procedures with delivering appropriate health care problematic (Shih et al., 2007) especially where procedures were not always seen to be consistent with care policy (Corley et al., 2010; Lee et al., 2020). Some nurses reported concern about the risk of contamination from taking uniforms home to wash (Corley et al., 2010; Viswanathan et al., 2020).

##### Impact on visitors

One intervention common amongst infection control procedures was to impose face‐to‐face visitor restrictions and/or reduce staff contact with patients. Several authors reported visitor restrictions causing a negative impact on the provision and experience of care for patients, nurses and families (Chan, Leung, et al., 2006; Corley et al., 2010). These include dignity and compassion during the end of life care (Danielis & Mattiussi, 2020; Rosa et al., 2020; Viswanathan et al., 2020), the sense of isolation experienced by patients and families (Fan et al., 2020) and the difficulty in delivering family‐centred care for children (Chan, Leung, et al., 2006). Commentaries highlighted the impact of infection control procedures on the end of life care where personal items were now considered contaminated (Neville, 2020). The rapid isolation of patients to contain the virus was reported as leading to some patients and family members feeling uninformed (Fan et al., 2020). Others noted that whilst reducing multiple entries into a room or ward may help reduce risk of contamination and the need to don and doff PPE, this may further increase the isolated patients’ sense of disconnection (Fan et al., 2020).

#### 3.2.4 Theme 4: emotional challenges of care

We identified four sub‐themes that illustrated the emotional challenges of providing fundamental nursing care to patients in the context of a pandemic including: fear and anxiety; lack of staff support in work or the community; difficulties with interaction between staff, patients and their families; burden of care.

##### Fear and anxiety

Fear and anxiety were common feelings reported by healthcare staff in several studies including the fear of the unknown and the fear of contracting the disease. Fears arose partly due to uncertainty about the disease, but were also related to concerns about the adequacy of PPE (Chan, Leung, et al., 2006; Corley et al., 2010; Kang et al., 2018; Kim, 2018; Lee et al., 2020; Liu, Luo, et al., 2020; Shih et al., 2007; Shih et al., 2009). Staff reported fear and anxiety in relation to transmitting infection to colleagues, family and friends (Kang et al., 2018; Liu, Luo, et al., 2020; Shih et al., 2007; Viswanathan et al., 2020). These observations and experiences are also supported by commentary evidence (Adams, 2020; Chochinov et al., 2020; Fedele, 2020; Rosa et al., 2020). Uncertainty about how best to care for patients, and fears about the risks of becoming infected created self‐doubt amongst staff and left some hesitant about wanting to be near or care for patients (Kim, 2018; Liu, Zhai, et al., 2020).

##### Lack of support in the workplace or in the community

Authors of several research studies reported that nurses value comradeship with their colleagues, but a lack of support in their work place (Kang et al., 2018; Kim, 2018) left them feeling unappreciated (Kim, 2018). Other authors reported that staff felt isolated and alienated due to their place and context of work (Kim, 2018; Lee et al., 2020). Nurses working on isolation wards reported feeling stigmatized by their peers and society, sometimes accentuated by the media (Lee et al., 2020).

##### Interaction between patients, staff and family members

In addition to the role of family members being already restricted during pandemic situations (Kim, 2018; Lee et al., 2020), authors of several research studies reported further challenges that staff face in communicating and interacting with patients and their families (Liu, Luo, et al., 2020; Rosa et al., 2020; Shih et al., 2007). This included the difficulties of using technology in maintaining good communication in stressful situations (Liu, Luo, et al., 2020) and sharing difficult news and end of life experiences with families (Rosa et al., 2020). The combined effect of these measures was reported as leading to patients being left alone between ward rounds with no‐one to comfort them (Andertun et al., 2017), psychological issues developing for patients (Liu, Luo, et al., 2020) and staff needing to deal with the emotional aftermath for patients of limited contact with their families (Danielis & Mattiussi, 2020). Concerns relating to dying with dignity and dying alone were also raised as an area of concern for staff and families during the COVID‐19 pandemic (Danielis & Mattiussi, 2020).

##### Burden of care

The unfamiliarity of infection care work, wearing of protective gear and lack of necessary skills in using new equipment added to the burden of nursing care (Lee et al., 2020). Some research reported that wards used to care for and isolate patients with SARS were improvised from any space available around the hospital. This sometimes meant they did not meet the criteria for infectious disease units and often lacked appropriate equipment. As a result, nurses felt that they could not do enough for the patient (Chan, Leung, et al., 2006; Liu & Liehr, 2009).

Evidence from previous pandemics suggests that nursing staff often feel an inability to meet patients’ needs (Chan, Leung, et al., 2006; Lee et al., 2020; Viswanathan et al., 2020) and, in particular, that they are failing to protect their patients’ dignity while dying (Lee et al., 2020). Some nurses reported the challenges they felt from dealing with the ‘same type’ of patients (patients with the same high level care needs) as they acknowledged these patients required additional emotional support, which left nurses feeling ‘burned out’ (Corley et al., 2010). Other studies reported the counter‐intuitive feelings of having to put their own or their colleagues’ safety above the needs of their patients (Andertun et al., 2017; Liu, Luo, et al., 2020), the guilt of those unable to work while their colleagues were over‐burdened and the emotional burden of the constant barrage of news and conversations about the pandemic (Viswanathan et al., 2020).

Commentaries suggest similar experiences are happening during the COVID‐19 pandemic with barriers to fulfilling fundamental care needs reported from several sources (Cathcart, 2020; Cena et al., 2020; Chochinov et al., 2020; Danielis & Mattiussi, 2020). There have been concerns with the lack of emotional support that nursing staff are able to offer patients and their families, and where the increasing strain on the health care system may mean that decisions about patient care are based on resource and protection of others, rather than being patient‐centred (Danielis & Mattiussi, 2020; Feder et al., 2020; Viswanathan et al., 2020). Commentaries also describe staff burnout, moral distress (Rosa et al., 2020), dealing with patient anxiety (Fan et al., 2020), and a lack of formal and informal peer support for nursing staff as a result of structural changes and the loss of dedicated spaces to promote community in the hospital environment (Hofmeyer et al., 2020a).

### 3.3 Adaptations

Four areas of potential adaptations were identified that cut across the barriers highlighted above: communication, organization of care, support for nursing staff and nurse leadership.

#### 3.3.1 Communication

Several research studies highlighted the importance of communication between nurse leaders and other nursing staff, between staff and patients, and staff and their patients’ significant others. Communication was described as key for sharing burden, as well as being able to interact with patients and their families and meet patients’ needs, particularly when staff were undertaking new roles (Hart et al., 2020; Shih et al., 2009).

Authors of commentaries highlighted several approaches to counteract communication difficulties caused by wearing PPE, ranging from putting photos of staff faces on aprons and visors, putting smiles on facemasks or exaggerating non‐verbal communication (Brown‐Johnson et al., 2020; Pettis, 2020). Further examples include staff communicating with patients and colleagues using whiteboards, blackboards, intercoms, cards and post it notes (Bagnasco et al., 2020; Fedele, 2020). One commentary described sharing expertise from palliative care by using the Patient Dignity Question (PDQ) which asks patients ‘What do I need to know about you as a person to take the best care of you possible?’ to promote empathy and connectedness with patients (Chochinov et al., 2020).

Others reported using technology to allow contact with families and psychological care services (Neville, 2020; Pettis, 2020; Tsai et al., 2020). Similar suggestions relating to the use of technology also included establishing a communication plan with patients and family members that sets out who will be involved and when and to identify and mitigate any barriers (Hart et al., 2020); allowing patients to see their health care plan to monitor results of tests and enable patients to make requests and ask questions to the nursing team (via a tablet or similar) (Fan et al., 2020); using video‐conferencing with interpretation services (Diamond et al., 2020; Hart et al., 2020; Humphreys et al., 2020); ensuring a bedside telephone to enable patients to contact their families where more advanced technology is not available or appropriate (Fan et al., 2020) and highlighting the importance of preparing families before seeing their critically ill loved ones by spending time describing the patient's visual condition before establishing a video connection with the patient (Kuntz et al., 2020). However, some commentators suggested that it is not feasible to deliver (or receive) comprehensive instruction around telehealth and communication in a pandemic situation (Hart et al., 2020).

Where face‐to‐face communication is not possible, it is suggested that other practices can build on and improve relationships between patients and staff, such as the improvement of patient and family education resources to ensure new knowledge builds on existing understanding (Tiwari et al., 2003); the use of daily communication to keep family members updated (Chan, Leung, et al., 2006); sharing information about the disease and care plans with staff, patients or family to ensure any concerns are dealt with (Shih et al., 2007); making provisions for face to face visiting at the end of life (Estella, 2020); ensuring patients and their family receive clear explanation of any restrictive policies that limit the physical presence of family members (Hart et al., 2020); using language and tone that seek to defuse and avoid conflict and using public information materials that empower patients and families to anticipate and prepare for next steps (Hart et al., 2020). Several guides for communication have been developed to improve communication between staff and patients at the end of life (DeCastro et al., 2020; Feder et al., 2020; Hart et al., 2020) and provide suggested language for non‐palliative specialist clinicians to conduct difficult critical conversations with patients and families during the COVID‐19 pandemic.

#### 3.3.2 (Re‐) Organization of care

Adaptations to the organization of care are reported to have a positive impact on the physical health and mental wellbeing of nurses as well as their workload, their ability to control contamination and their interactions with each other, their patients and patients’ families.

Some research highlights how alternative models of staffing have been used to address care. These include a ‘two‐by two’ rule, under which nurses caring for patients with SARS did everything in pairs, assuring that a nurse always had someone to turn to for assistance (Liu & Liehr, 2009); ‘modular care’ to promote patient‐focused care by enabling health care staff to spend more time getting to know their patient and provide continuity of care (Chan et al., 2008; Chan, Chung, et al., 2006); co‐ordinating care or communications with patients so that times when the nurse planned to be in the room for patient care could also be used to undertake other tasks such as introducing or removing items from the room and participation in meetings with either other care staff/teams or family communication (Kuntz et al., 2020). Another example is a data‐based nursing care model used with paediatric patients with suspected SARS, striking a balance between attending to the physical and psychological needs of the child and adhering to precautions for combating disease spread (Chan, Leung, et al., 2006). This study introduced an early discharge policy where for a suspected SARS patient a more speedy discharge decision was made once the child had a good response to treatment, or tests were negative (Chan, Leung, et al., 2006). Commentary evidence suggested that sharing expertise across specialties to enable care teams to deliver the most appropriate care, for example a palliative unit nurse providing in‐service training to nurses on a COVID‐19 ward (Pahuja & Wojcikewych, 2020) or determining the most appropriate tasks for team members with and without critical care training, helps tackle problems with lack of experience (Martland & Huffines, 2020).

Other organizational adaptations included introducing a higher nurse patient ratio, providing more breaks while on duty and scheduling a shorter working week (Tiwari et al., 2003); increasing ancillary staff levels to cope with extra cleaning duties, extra waste management, patient repositioning and transfers (Corley et al., 2010); amalgamating activities and designating a nurse to be a ‘runner’ to retrieve supplies or medications, thereby preventing the need for staff to don and doff PPE (Newby et al., 2020) and providing staff with hot meals in disposable trays, snacks and comfort food directly to the wards (Cintoni et al., 2020). Also suggested were organizing staff huddles to discuss team assignments, patient care goals and red flags that should be reported immediately at the start of and at regular intervals throughout a shift which are also thought to support better communication between staff and staff teams (Cathcart, 2020; Martland & Huffines, 2020).

Further examples included physical reorganization of the hospital environment such as increasing space between beds, between staff and between patients; providing patients with bottled water, and not allowing family to bring in home‐cooked food (Tiwari et al., 2003); the introduction of a ‘no‐touch’ policy to reduce the amount of the infectious virus on PPE (Liu & Liehr, 2009); allowing theatre scrubs to be worn while working in the isolated unit and laundering uniforms in the hospital (Corley et al., 2010; Viswanathan et al., 2020). Further adaptations suggested include small changes such as providing personalized meal provision to meet the increased energy and protein requirements of patients who are able to eat while supporting those unable to eat with nutritional formulas (Cintoni et al., 2020). Staff changing their routines, such as showering before leaving the hospital, wearing easy to clean rubber shoes (Fedele, 2020) and staying in hotel or rental accommodation (Viswanathan et al., 2020) have also been suggested.

Other commentaries reflected on how the use of technology (for example tablet computers) may ease some of the pressure on health care staff by reducing the number of individuals entering a patient's room to undertake physiological monitoring and enabling an electronic signature system on a tablet (Hart et al., 2020; Humphreys et al., 2020; Newby et al., 2020; Pahuja & Wojcikewych, 2020; Tsai et al., 2020; Umoren et al., 2020).

Adaptations that focus more on the organization or experience of the end of life care included facilitating family visits by educating and supporting a single designated family member, thus ensuring a balance between infection prevention control policies and family‐centred care (Bouchoucha & Bloomer, 2020) and working with infection control teams to create innovative framed reminders of deceased loved ones, such as fingerprints or print outs of electrocardiograms (Neville, 2020).

#### 3.3.3 Support for staff

Providing appropriate levels of care and training for nurses themselves by ensuring staff are physically and mentally well and have the knowledge and confidence to deliver the best care possible can break down many barriers to patient care.

Some research highlighted mechanisms to improve support for staff in the workplace including holding daily meetings between senior medical and nursing staff which provide a forum to problem solve and feedback issues to each other (Corley et al., 2010) and other group‐based interventions to share experiences in a peer setting, learn from peers and build a sense of solidarity and camaraderie (Viswanathan et al., 2020). The medical and nursing management team can ensure extra measures to show appreciation for the hard work of staff, e.g. by providing food, such as pizza, chocolates and fruit platters and messages of thanks (Corley et al., 2010). Furthermore, acknowledging the importance of staff caring for each other on the ward (Liu & Liehr, 2009) and providing self‐care tips such as encouraging minibreaks, brief mental relaxation or short meditation strategies, physical exercise, sleep, healthy nutrition, recreation and reducing media contact (Fedele, 2020; Viswanathan et al., 2020) can also help staff to fee supported.

Several commentaries also promoted the idea of psychological support to enable staff to talk about concerns or fears and support each other, either via mental health professionals or pastoral supporters (Bagnasco et al., 2020; Cathcart, 2020; Hofmeyer et al., 2020a, 2020b; Martland & Huffines, 2020; Morley et al., 2020). Others highlighted the need for staff leaders to be proactive in anticipating potentially difficult/challenging situations (Morley et al., 2020) to prevent staff burnout, encourage self‐care and wellbeing and promote strategies for dealing with moral or psychological distress, safe use of PPE and maintaining a healthy work environment (Hofmeyer et al., 2020b; Martland & Huffines, 2020). Some mentioned taking time to honour patients by stopping work and standing in silence for a short time to acknowledge the death of a patient (Cathcart, 2020) or describe the use of ‘Schwartz rounds’ that bring clinical and non‐clinical staff together to discuss the psychological and emotional impact of caring for patients and families (Hofmeyer et al., 2020a).

Authors reporting interview studies of nurses caring for patients with SARS (Liu & Liehr, 2009) and MERS (Kang et al., 2018) described the importance of empirical knowledge about infectious diseases in general and specifically the risk of contagion in enabling nurses to meet complex patient needs. Establishing consistent and solid practice guidelines early on, sharing them clearly (Kang et al., 2018; Liu & Liehr, 2009), training health care workers to deliver the information and building consensus among team members about infection control procedures (Shih et al., 2007) were also highlighted as important and valued approaches. Training that includes examples from real‐life situations and the hands‐on knowledge of staff with previous experience of pandemic situations was described as helpful with the additional benefit of driving a sense of healing for those sharing their experiences (Holmgren et al., 2019). Several commentaries discussed how information could be better collated and distributed so that the burden of keeping up to date with constantly changing guidelines and practices was reduced to a more consistent communication chain (Adams, 2020; Martland & Huffines, 2020).

#### 3.3.4 Leadership

Several authors reflected on the best management style for nurse leaders during a pandemic and reported how leadership can affect communication, wellbeing, knowledge and feelings of support.

One study highlighted the importance of a supportive informed leader. The qualities of a good leader in this context incorporated listening, professionalism, calmness, experience, effectiveness, encouragement, empathy, social competence, support, information and objectivity (Holmgren et al., 2019). Other leadership skills designed to empower nurses to work as experts and positively influence the quality of care pertain to the organization of care, being confident in making changes to optimize workflow (Tsai et al., 2020), and acknowledging nurses’ work as valuable (Kang et al., 2018). Commentaries on the COVID‐19 pandemic highlighted the need for nurse leaders to promote a healthy work environment and reinforce staff resilience. Suggestions included actively and meaningfully engaging in communication, recognizing their efforts and burden of care (Martland & Huffines, 2020), collaborating with staff to identify ways to acknowledge and reward effort, promoting flexible, family friendly work environments that meet the needs of a diverse workforce, understanding what motivates their staff to do their best work, supporting work‐life integration and recommending trusted sources to support self‐protection and maintain wellbeing (Hofmeyer et al., 2020a).

Having been able to use the synthesis to answer our first research question and identify several potential adaptations to providing fundamental nursing care, we hoped also to be able to find evidence to help answer our second research question.

### 3.4 Question 2: how have adaptations to fundamental nursing care practices as a result of COVID‐19 and other conditions requiring isolation impacted overall patient experience, care quality, functional ability and treatment outcomes for patients with COVID‐19 in hospital?

However, our searches did not identify any literature that specifically investigated the impact of any adaptations on our outcomes of interest—overall patient experience, care quality, functional ability or treatment outcomes for patients. Three research studies described the *use* of adaptations such as the use of telemedicine for family interactions (Kuntz et al., 2020), ward rounds (Umoren et al., 2020), or a change in the organization of care on the ward (Chan et al., 2008), with one study describing an intervention to support nurses (Viswanathan et al., 2020). Although these studies do not report on our outcomes of interest, one does suggest that the use of technology to communicate with families (E‐Family meeting) enabled families to understand the condition and needs of their loved one whilst in hospital and to build their trust in the clinical team (Kuntz et al., 2020). Using this type of technology was also reported to reduce the use of PPE (Kuntz et al., 2020; Umoren et al., 2020). Another study which looked at a change in the organization of care (modular care) (Chan et al., 2008) suggests that 54% of nurses reported improvements in nurse‐patient interaction resulting from perceived improvements in continuity of care, 26% of nurses believed their work became more efficient and 38% believed that the organizational adaptation had resulted in enhanced infection control. The same study reported using a generic patient satisfaction scale to score the patients’ overall experience of care during the organizational change, no statistically significant differences in satisfaction were reported but it was proposed that nurse‐patient ratios influenced patients’ expectations of nurses (Chan et al., 2008).

Finally, to inform our third research question, we used the literature identified in this review to produce a summary of the available protocols, guidance and research related to specific aspects of fundamental care during a pandemic.

### 3.5 Question 3: what are the areas of fundamental nursing care, for patients with COVID‐19 in hospital, that are evident/missing in published protocols and guidance?

Table 8 provides a summary of the literature identified in this review, enabling us to see how each of the areas of fundamental nursing care is covered by existing research, protocols or guidelines, including commentary pieces and where the gaps remain. Gaps are identified in the following areas of fundamental nursing care: personal cleansing, eating and drinking, rest and sleep, mobility, medication management, non‐verbal communication with patients, shared decision making with patients, dignity and respect needs and respecting values and beliefs. Four of the 17 areas of fundamental care have relatively substantial levels of research input and guideline development (nurse support, organization of care, communication with patients and relatives and patient safety). Conversely, for seven of the 17 areas of fundamental nursing care (toileting needs, mobility, medication management, establishing relationships with patients, non‐verbal communication with patients, dignity and respect and respecting values and beliefs), we were unable to identify any evidence of guidelines or protocols. Of these seven, three areas (mobility, medication management and non‐verbal communication with patients) also had no research evidence to support them.

**TABLE 8:** Gaps in fundamental nursing care (FNC) during a pandemic

	Fundamental care area^a^	Protocols/guidance	Adapting/reorganizing care	Experience of care/Barriers to giving FNC	Patient experience
**Physical**	Global Physical	**3** Evidence‐based & Consensus Guidance	7 Guidance, Quant, Qual, Commentary	**9** Quant, Qual, Commentary	—
	Personal cleansing (including oral/mouth care) and dressing	**2** Evidence‐based Guidance	—	—	—
	Toileting needs	—	**1** Quant	**3** Quant, Qual	—
	Eating and drinking	**8** Protocols, Evidence & Consensus Guidance, Commentary	**3** Commentary	**5** Qual, Review, Commentary	—
	Rest and sleep	1 Evidence‐based Guidance	**1** Review	**1** Review	—
	Mobility	—	—	—	—
	Comfort (pain management, breathing easily and temperature control)	6 Protocols, Evidence & Consensus Guidance, Commentary	**1** Commentary	**4** Guidance, Qual, Review, Commentary	—
	Safety (risk assessment and management, infection prevention and minimizing complications)	2 Consensus Guidance, Commentary	**14** Quant, Qual, Commentary	**19** Guidance, Quant, Qual, Review, Commentary	**1** Qual
	Medication management	1 Commentary	**4** Commentary	**3** Commentary	—
**Relational**	Establishing a relationship with patients	—	**13** Quant, Qual, Commentary	**13** Quant, Qual, Review, Commentary	—
	Talking and listening to patients	**2** Consensus Guidance	6 Quant, Qual, Commentary	**7** Quant, Qual, Commentary	—
	Non‐verbal communication with patients	—	5 Commentary	**2** Commentary	—
	Shared decision making with patients	**2** Protocols	4 Commentary	**3** Review, Commentary	—
	Communicating with patients’ relatives, carers and significant others	**5** Expert & Consensus Guidance	**18** Quant, Qual, Commentary	**16** Quant, Qual, Review, Commentary	—
	Dignity and respect needs	—	7 Qual, Commentary	7 Qual, Commentary	—
	Respecting values and beliefs	—	5 Quant, Qual, Commentary	4 Quant, Qual, Commentary	—
	Emotional wellbeing and anxiety and low mood	2 Consensus Guidance	8 Guidance, Quant, Qual, Commentary	9 Quant, Qual, Commentary	**1** Qual
	Organization of care	1 Evidence‐based Guidance	**19** Quant, Qual, Commentary	**14** Quant, Qual, Commentary	**1** Qual
	Nurse Support	9 Evidence‐based & Consensus Guidance, Quant, Qual, Commentary	20 Guidance, Quant, Qual, Commentary	**17** Quant, Qual, Commentary	**1** Qual

Abbreviations: FNC, fundamental nursing care; Qual, qualitative research study;Quant, quantitative research study; Review, literature review but not a systematic review.

^a^ Aspects of fundamental care as defined by Kitson et al. (2010). Defining the fundamentals of care. *Int J Nurs Pract*, *16*, 423–434. The number in each cell refers to the total number of pieces of literature in this review that applied to that domain similarly the colour of each cell refers to the amount of literature on one particular domain as follows: 0 = 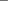, 1–5 = 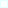, 6–10 = 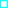, 11–15=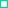, >15 = 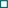.

We identified eight care protocols that gave updated guidance for aspects of fundamental care during the COVID‐19 pandemic. Of these, four focused on nutrition practices (Aguila et al., 2020; Caccialanza, 2020; Cena et al., 2020; Chapple et al., 2020), three on the re‐organization of care and/or nurse support (Buheji & Buhaid, 2020; Fausto et al., 2020; Maben et al., 2020) and one on communication practices with patients and families (deLima Thomas et al., 2020) (see Table 3). This highlights how few areas of fundamental nursing care have been the focus of new or updated guidelines. One of the most notable areas in this table is the lack of any research evidence or commentaries that take into account patients’ perspectives of their experience of fundamental nursing care or any of the adaptations that are made to improve the delivery of this care.

## 4 DISCUSSION

In this review, we located, appraised and synthesized 64 publications—19 data‐based research articles and 45 commentary pieces, protocols, reviews or guidelines. We derived four main themes and 11 sub‐themes on the barriers to fundamental care reported by nurses when caring for patients with the SARS‐COV‐2 virus or other pandemic infections. These barriers were addressed by multiple adaptations to care. We found that there was very little empirical evidence for the effect of these adaptations on patient experience, care quality, functional ability and treatment outcomes. The quantitative evidence, whether in primary research reports or guidelines, was often poor in quality but the qualitative studies were generally well conducted. There were very few guidelines specific to the fundamental care of patients with COVID‐19; those that did focussed on nutrition, organization of care, nurse support and communicating with patients’ significant others. Of particular note was the lack of studies collecting data from patients’ perspectives.

Barriers identified in the literature included working in PPE, staffing levels and new roles, infection control procedures and emotional challenges of patient care. Adaptions to these barriers ranged from enhanced communication strategies including the use of information technology, the re‐organization of care procedures or environments, strategies to support the mental health and wellbeing of nurses, and leadership and management actions.

The findings of this review are consistent with other recent research, highlighting similar barriers experienced by nurses in caring for patients with COVID‐19 (Barello et al., 2020; Joo & Liu, 2021). The impact of these barriers on nursing staff and the lack of hospital preparedness for delivering nursing care during a pandemic is real, and calls remain to encourage healthcare organizations to have systems in place that can support staff and give them confidence in working during pandemic and other health care emergencies (Manzano García & Ayala Calvo, 2021). Furthermore, the need for nurse leaders to be proactive in hearing, protecting, preparing, supporting and caring for their staff is also reiterated (Hofmeyer & Taylor, 2021). However, despite the proliferation of research (and commentary) on the barriers to and experiences of delivering (but not receiving) nursing care in a pandemic, there remains a vast gap in research to understand the effectiveness of interventions or adaptations that might help. The implications for nursing education are that educators and students do not have clear guidelines and materials with which to prepare themselves for practice in these situations.

The limitations of this review are largely due to the limitations of the available literature. Despite thorough searches conducted in several databases and multiple forms of supplementary searching, at the time of searching only 19 empirical studies were found, surprisingly few for an area of care that is so essential to patient experience and that covers such a broad range of care elements. Studies were not excluded based on their quality but the poor quality of the few quantitative studies available must be taken into account for future research and implementation work. Our decision to include English language only studies was taken as a result of time and resource restrictions and may mean some experiences were not included in this synthesis. Our inclusion of commentaries in this review is unusual but we wanted to be able to capture the experiences of and adaptations to care that might also be prominent or developing in the COVID‐19 pandemic and might not yet have been reported in established research literature. The commentaries cannot be considered with similar confidence as the research. Although there are some limitations to using an index paper to guide analyses, our approach, although deductive, was also responsive to capture the themes identified in the literature and the index paper was used as an initial ‘scaffold’ for the analysis once data had been collected rather than guiding the data collection itself. We also note that given the need to develop clinical guidelines for the large majority of patients with COVID‐19 who remain conscious during their admissions, our review did not include the nursing care of unconscious invasively intubated patients.

More high‐quality research is needed to understand the real impact of the many adaptations found in this review on the experience of patients’ and healthcare staff, and on the organization and delivery of care. Consequently, the themes and evidence summaries identified in this review (along with data from the survey and consensus groups from the wider study) have been used to develop a pandemic‐specific fundamental nursing care protocol (ISRCTN 13177364) (Richards et al., 2021) which we are now testing in a cluster randomized controlled trial. This trial will be the first such empirical test of many of the suggestions made by researchers, clinical nurses and commentators in the reports cited in our review here. There is, nonetheless, room for many more studies into the impact of these strategies, individually or combined, on patient experience, care quality, functional ability and treatment outcomes.

## 5 CONCLUSION

The emergence of the SARS‐COV‐2 virus (COVID‐19) has both highlighted the importance of the nursing profession and how under‐prepared and under‐supported healthcare staff have been during the 2020/2021 pandemic. Nurses have had to adapt their care, learn new skills and prepare for situations they have never experienced before. Our review highlights the particular barriers faced in maintaining the delivery of fundamental care for patients with COVID‐19 and draws attention to some of the adaptations that might be helpful to work through these barriers. However, the research behind these adaptations is lacking and therefore the predicted improvements in the delivery and experience of care and support for both patients and health care staff remain uncertain. To be prepared for the ongoing COVID‐19 pandemic and any future pandemics, research on adaptations to healthcare delivery and staff support must be prioritized to understand what will enable healthcare services to respond quickly and confidently in similar situations in the future.

## CONFLICT OF INTEREST

No conflict of interest has been declared by the authors.

## AUTHOR CONTRIBUTIONS

All authors have agreed on the final version and meet at least one of the following criteria (recommended by the ICMJE): (1) substantial contributions to conception and design, acquisition of data, or analysis and interpretation of data; (2) drafting the article or revising it critically for important intellectual content.

JTC, DAR, RA, RW and AB consulted with co‐authors HVRS, AMRu, HI‐S, EC, SC, GJM‐T, PL, AMRa, MS and ST, the remaining project co‐investigators and the patient advisory group to devise the review and gain feedback on the protocol including search terms and sources and interpretation of the findings. AB conducted the literature searches and document retrieval. JTC, RA, and RW completed screening, data extraction and quality appraisal. JTC, BA and RW with the support of RG synthesised the results. RW, JTC, RA, DAR, AB and RG drafted the manuscript. All other authors reviewed the manuscript and made editorial suggestions. All authors read and approved the final manuscript.

### PEER REVIEW

The peer review history for this article is available at https://publons.com/publon/10.1111/jan.15047.

## Supporting information

Appendix A

Appendix B

## Data Availability

Data sharing not applicable—no new data generated.

## References

[jan15047-bib-0001] Adams, C. (2020). Goals of care in a pandemic: Our experience and recommendations. Journal of Pain and Symptom Management, 60(1), e15–e17. 10.1016/j.jpainsymman.2020.03.018 PMC7270987 32240752

[jan15047-bib-0002] Aguila, E. J. T. , Cua, I. H. Y. , Fontanilla, J. A. C. , Yabut, V. L. M. , & Causing, M. F. P. (2020). Gastrointestinal manifestations of COVID‐19: Impact on nutrition practices. Nutrition in Clinical Practice, 35, 800–805. 10.1002/ncp.10554 32668037 PMC7405319

[jan15047-bib-0003] Aiken, L. H. , Sloane, D. , Griffiths, P. , Rafferty, A. M. , Bruyneel, L. , McHugh, M. , Maier, C. B. , Moreno‐Casbas, T. , Ball, J. E. , Ausserhofer, D. , & Sermeus, W. (2017). Nursing skill mix in European hospitals: Cross‐sectional study of the association with mortality, patient ratings, and quality of care. BMJ Quality & Safety, 26, 559–568. 10.1136/bmjqs-2016-005567 PMC5477662 28626086

[jan15047-bib-0004] Anderson, L. (2020a). Ensuring patients with Covid‐19 receive good nutritional care. Nursing times, 116, 34.

[jan15047-bib-0005] Anderson, L. (2020b). Providing nutritional support for the patient with COVID‐19. British Journal of Nursing, 29(8), 458–459. 10.12968/bjon.2020.29.8.458 32324465

[jan15047-bib-0006] Andertun, S. , Hornsten, A. , & Hajdarevic, S. (2017). Ebola virus disease: Caring for patients in Sierra Leone—A qualitative study. Journal of Advanced Nursing, 73(3), 643–652. 10.1111/jan.13167 27747916

[jan15047-bib-0007] Beck, M. , Antle, B. J. , Berlin, D. , Granger, M. , Meighan, K. , Neilson, B. J. , Shama, W. , Westland, J. , & Kaufman, M. (2004). Wearing masks in a pediatric hospital: developing practical guidelines. Canadian Journal of Public Health. Revue Canadienne de Sante Publique, 95(4), 256–257. 15362465 10.1007/BF03405126 PMC6976022

[jan15047-bib-0008] Bagnasco, A. , Zanini, M. , Hayter, M. , Catania, G. , & Sasso, L. (2020). COVID 19—A message from Italy to the global nursing community. Journal of Advanced Nursing, 76(9), 2212–2214. 10.1111/jan.14407 32352175 PMC7267658

[jan15047-bib-0009] Barello, S. , Falcó‐Pegueroles, A. , Rosa, D. , Tolotti, A. , Graffigna, G. , & Bonetti, L. (2020). The psychosocial impact of flu influenza pandemics on healthcare workers and lessons learnt for the COVID‐19 emergency: A rapid review. International Journal of Public Health, 65(7), 1205–1216. 10.1007/s00038-020-01463-7 32888048 PMC7472941

[jan15047-bib-0010] Baumann, A. , Blythe, J. , Underwood, J. , & Dzuiba, J. (2003). Capacity, casualization, and continuity: The impact of SARS Toronto, Canada.

[jan15047-bib-0011] Bethel, A. C. , Rogers, M. , & Abbott, R. (2021). Use of a search summary table to improve systematic review search methods, results, and efficiency. Journal of the Medical Library Association, 109(1), 97–106. 10.5195/jmla.2021.809 33424470 PMC7772975

[jan15047-bib-0012] Black, N. , Varaganum, M. , & Hutchings, A. (2014). Relationship between patient reported experience (PREMs) and patient reported outcomes (PROMs) in elective surgery. BMJ Quality & Safety, 23, 534–542. 10.1136/bmjqs-2013-002707 24508681

[jan15047-bib-0013] Bouchoucha, S. , & Bloomer, M. J. (2020). Family‐centred care during a pandemic: The hidden impact of restricting family visits. Nursing & Health Sciences, 13, 13. 10.1111/nhs.12748 PMC7323067 32533617

[jan15047-bib-0014] Brown‐Johnson, C. , Vilendrer, S. , Heffernan, M. B. , Winter, S. , Khong, T. , Reidy, J. , & Asch, S. M. (2020). PPE portraits‐a way to humanize personal protective equipment. Journal of General Internal Medicine, 35(7), 2240–2242. 10.1007/s11606-020-05875-2 32410125 PMC7224350

[jan15047-bib-0015] Buheji, M. , & Buhaid, N. (2020). Nursing human factor during COVID‐19 pandemic. International Journal of Nursing, 10(1), 12–24. 10.1016/j.aucc.2020.06.002

[jan15047-bib-0016] Bureau of Health Information . (2014). Adult Admitted Patient Survey 2013 Results. Snapshot Report NSW Patient Survey Program. https://www.bhi.nsw.gov.au/BHI_reports/patient_survey_results/adult_admitted_patient_survey_2013_results#main_report

[jan15047-bib-0017] Caccialanza, R. , Laviano, A. , Lobascio, F. , Montagna, E. , Bruno, R. , Ludovisi, S. , Corsico, A. G. , Di Sabatino, A. , Belliato, M. , Calvi, M. , Iacona, I. , Grugnetti, G. , Bonadeo, E. , Muzzi, A. , & Cereda, E. (2020). Early nutritional supplementation in non‐critically ill patients hospitalized for the 2019 novel coronavirus disease (COVID‐19): Rationale and feasibility of a shared pragmatic protocol. Nutrition, 74, 10.1016/j.nut.2020.110835 PMC7194616 32280058

[jan15047-bib-0018] Canadian Nurses Association . (2003). CNA Brief to the National Advisory Committee on SARS and Public Health: Lessons learned and recommendations. https://www.cna‐aiic.ca/‐/media/cna/page‐content/pdf‐en/cna_brief_national_advisory_committee_sars_e.pdf?la=en&hash=12F9C0D89634D9B506E707EBFF8708E171F01D19

[jan15047-bib-0019] Cathcart, E. B. (2020). The new nurse manager survival guide, part II. Nursing Management, 51(6), 17. 10.1097/01.NUMA.0000662704.97080.df PMC7258746 32379170

[jan15047-bib-0020] Cena, H. , Maffoni, S. , Braschi, V. , Brazzo, S. , Pallavicini, C. , Vietti, I. , Portale, S. , & Corradi, E. (2020). Position paper of the Italian association of medical specialists in dietetics and clinical nutrition (ANSISA) on nutritional management of patients with COVID‐19 disease. Mediterranean Journal of Nutrition and Metabolism, 13(2), 113–117. 10.3233/MNM-200425

[jan15047-bib-0021] Chan, E. A. , Chung, J. W. , & Wong, T. K. (2008). Learning from the severe acute respiratory syndrome (SARS) epidemic. Journal of Clinical Nursing, 17(8), 1023–1034. 10.1111/j.1365-2702.2007.01997.x 18179533

[jan15047-bib-0022] Chan, E. A. , Chung, J. W. , Wong, T. K. , & Yang, J. C. (2006). An evaluation of nursing practice models in the context of the severe acute respiratory syndrome epidemic in Hong Kong: A preliminary study. Journal of Clinical Nursing, 15(6), 661–670. 10.1111/j.1365-2702.2007.01997.x 16684161

[jan15047-bib-0023] Chan, S. S. , Leung, D. Y. , Wong, E. M. , Tiwari, A. F. , Wong, D. C. , Lo, S. L. , & Lau, Y. L. (2006). Balancing infection control practices and family‐centred care in a cohort of paediatric suspected severe acute respiratory syndrome patients in Hong Kong. Journal of Paediatrics and Child Health, 42(1–2), 20–27. 10.1111/j.1440-1754.2006.00776.x 16487385

[jan15047-bib-0024] Chapple, L.‐A. , Fetterplace, K. , Asrani, V. , Burrell, A. , Cheng, A. C. , Collins, P. , Doola, R. , Ferrie, S. , Marshall, A. P. , & Ridley, E. J. (2020). Nutrition management for critically and acutely unwell hospitalised patients with coronavirus disease 2019 (COVID‐19) in Australia and New Zealand. Australian Critical Care, 33(5), 399–406. 10.1016/j.aucc.2020.06.002 32682671 PMC7330567

[jan15047-bib-0025] Cheng, S. K. W. , Wong, C. W. , Wong, M. , Chang, S. , Wong, K. C. , Chong, G. , & So, S. (2005). Therapeutic responses of health‐care workers to patients with Severe Acute Respiratory Syndrome (SARS) in the acute phase. Asian Journal of Nursing Studies, 8(3), 2–8.

[jan15047-bib-0026] Chochinov, H. M. , Bolton, J. , & Sareen, J. (2020). Death, dying, and dignity in the time of the COVID‐19 pandemic. Journal of Palliative Medicine, 23(10), 1294–1295. 10.1089/jpm.2020.0406 32639895 PMC7523014

[jan15047-bib-0027] Cintoni, M. , Rinninella, E. , Annetta Maria, G. , & Mele, M. C. (2020). Nutritional management in hospital setting during SARS‐CoV‐2 pandemic: A real‐life experience. European Journal of Clinical Nutrition, 74(5), 846–847. 10.1038/s41430-020-0625-4 32253375 PMC7135971

[jan15047-bib-0028] Corley, A. , Hammond, N. E. , & Fraser, J. F. (2010). The experiences of health care workers employed in an Australian intensive care unit during the H1N1 Influenza pandemic of 2009: A phenomenological study. International Journal of Nursing Studies, 47(5), 577–585. 10.1016/j.ijnurstu.2009.11.015 20036360 PMC7125717

[jan15047-bib-0029] Critical Appraisal Skills Programme . (2018). CASP Qualitative checklist. https://casp‐uk.net/casp‐tools‐checklists/

[jan15047-bib-0030] Danielis, M. , & Mattiussi, E. (2020). The care of patients through the lens of the fundamentals into times of the COVID‐19 outbreak. Intensive and Critical Care Nursing, 60, 102883. 10.1016/j.iccn.2020.102883 32448629 PMC7241975

[jan15047-bib-0031] Darzi, A. (2008). Quality and the NHS next stage review. Lancet, 371, 1563–1564. 10.1016/S0140-6736(08)60672-8 18468532

[jan15047-bib-0032] DeCastro, M. S. N. , Zak, B. S. , Pccn, J. , & Thum, M. S. N. (2020). Nursing and provider safety checklist for Covid‐19 patients. Philadelphia University and Thomas Jefferson University. jdc.jefferson.edu

[jan15047-bib-0033] deLima Thomas, J. , Leiter, R. E. , Abrahm, J. L. , Shameklis, J. C. , Kiser, S. B. , Gelfand, S. L. , Sciacca, K. R. , Reville, B. , Siegert, C. A. , Zhang, H. , Lai, L. , Sato, R. , Smith, L. N. , Kamdar, M. M. , Greco, L. , Lee, K. A. , Tulsky, J. A. , & Lawton, A. J. (2020). Development of a palliative care toolkit for the COVID‐19 pandemic. Journal of Pain and Symptom Management, 60(2), e22–e25. 10.1016/j.jpainsymman.2020.05.021 PMC7255186 32454184

[jan15047-bib-0034] Department of Health . (2013). Report of the Mid Staffordshire NHS Foundation Trust Public Enquiry. https://www.gov.uk/government/publications/report‐of‐the‐mid‐staffordshire‐nhs‐foundation‐trust‐public‐inquiry

[jan15047-bib-0035] Diamond, L. C. , Jacobs, E. A. , & Karliner, L. (2020). Providing equitable care to patients with limited dominant language proficiency amid the COVID‐19 pandemic. Patient Education & Counseling, 103(8), 1451–1452. 10.1016/j.pec.2020.05.028 32571503 PMC7304953

[jan15047-bib-0036] Dingfield, L. E. , Flores, E. J. , Radcliff, J. A. , Stamm, R. , & Uritsky, T. J. (2020). Adapting a comfort care order set in a large health system during the COVID‐19 pandemic. Journal of Palliative Medicine, 23(8), 1004–1005. 10.1089/jpm.2020.0277 32429747

[jan15047-bib-0037] Doyle, C. , Lennox, L. , & Bell, D. (2013). A systematic review of evidence on the links between patient experience and clinical safety and effectiveness. British Medical Journal Open, 3(1), e001570. 10.1136/bmjopen-2012-001570 PMC3549241 23293244

[jan15047-bib-0038] Effective Public Health Practice Project . (1998). Quality assessment tool for quantitative studies. http://www.ephpp.ca/tools.html

[jan15047-bib-0039] Estella, A. (2020). Compassionate communication and end‐of‐life care for critically Ill patients with SARS‐CoV‐2 infection. Journal of Clinical Ethics, 31(2), 191–193. 32585665

[jan15047-bib-0040] Fan, P. E. M. , Fazila, A. , Lim, S. H. , Ang, S. Y. , Karen, P. , Quek, A. H. , Quek, H. K. S. , & Tracy, C. A. (2020). Needs and concerns of patients in isolation care units—Learnings from COVID‐19: A reflection. World Journal of Clinical Cases, 8(10), 1763–1766. 10.12998/wjcc.v8.i10.1763 32518768 PMC7262715

[jan15047-bib-0041] Fang, J. , Liu, Y. T. , Lee, E. Y. , & Yadav, K. (2020). Telehealth solutions for in‐hospital communication with patients under isolation during COVID‐19. Western Journal of Emergency Medicine, 21(4), 801–806. 10.5811/westjem.2020.5.48165 32726245 PMC7390554

[jan15047-bib-0042] Fausto, J. , Hirano, L. , Lam, D. , Mehta, A. , Mills, B. , Owens, D. , Perry, E. , & Curtis, J. R. (2020). Creating a palliative care inpatient response plan for COVID‐19‐the UW medicine experience. Journal of Pain & Symptom Management, 60(1), e21–e26. 10.1016/j.jpainsymman.2020.03.025 PMC7171263 32240754

[jan15047-bib-0043] Fedele, R. (2020). ‘It's surreal’: Nursing during the COVID‐19 pandemic. Australian Nursing and Midwifery Journal. https://anmj.org.au/its‐surreal‐nursing‐during‐the‐covid‐19‐pandemic/

[jan15047-bib-0044] Feder, S. , Akgün, K. M. , & Schulman‐Green, D. (2020). Palliative care strategies offer guidance to clinicians and comfort for COVID‐19 patient and families. Heart and Lung, 49(3), 227–228. 10.1016/j.hrtlng.2020.04.001 32307120 PMC7128096

[jan15047-bib-0045] Feo, R. , Conroy, T. , Jangland, E. , Muntlin Athlin, Å. , Brovall, M. , Parr, J. , Blomberg, K. , & Kitson, A. (2018). Towards a standardised definition for fundamental care: A modified Delphi study. Journal of Clinical Nursing, 27, 2285–2299. 10.1111/jocn.14247 29278437

[jan15047-bib-0046] Gammon, J. , & Hunt, J. (2018). A review of isolation practices and procedures in healthcare settings. British Journal of Nursing, 27, 137–140. 10.12968/bjon.2018.27.3.137 29412028

[jan15047-bib-0047] Garling, P. (2008). Final report of the Special Commission of Inquiry Acute Care Services in NSW Public Hospitals. https://www.dpc.nsw.gov.au/assets/dpc‐nsw‐gov‐au/publications/Acute‐Care‐Services‐in‐NSW‐hospitals‐listing‐437/052e7ca07b/First‐Report‐of‐the‐Special‐Commission‐of‐Inquiry‐into‐Acute‐Care‐Service‐in‐NSW‐Public‐Hospitals.pdf

[jan15047-bib-0048] Google . (2020). Google Jamboard. https://workspace.google.com/products/jamboard/

[jan15047-bib-0049] Graham, C. , Kasbauer, S. , Cooper, R. , King, J. , Sizmur, S. , Jenkinson, C. , & Kelly, L. (2018). An evaluation of a near real‐time survey for improving patients’ experiences of the relational aspects of care: A mixed‐methods evaluation. NIHR Journals Library. 29595933

[jan15047-bib-0050] Groven, F. M. V. , Zwakhalen, S. M. G. , Odekerken‐Schröder, G. , Joosten, E. J. T. , & Hamers, J. P. H. (2017). How does washing without water perform compared to the traditional bed bath: A systematic review. BMC Geriatrics, 17(1), 31. 10.1186/s12877-017-0425-4 28118815 PMC5264342

[jan15047-bib-0051] Hart, J. L. , Turnbull, A. E. , Oppenheim, I. M. , & Courtright, K. R. (2020). Family‐centered care during the COVID‐19 era. Journal of Pain & Symptom Management, 60(2), e93–e97. 10.1016/j.jpainsymman.2020.04.017 32333961 PMC7175858

[jan15047-bib-0052] Higgins, J. P. T. , Thomas, J. , Chandler, J. , Cumpston, M. , Li, T. , Page, M. J. , & Welch, V. A. (2020). Cochrane handbook for systematic reviews of interventions version 6.1. www.training.cochrane.org/handbook

[jan15047-bib-0053] Hofmeyer, A. , & Taylor, R. (2021). Strategies and resources for nurse leaders to use to lead with empathy and prudence so they understand and address sources of anxiety among nurses practising in the era of COVID‐19. Journal of Clinical Nursing, 30(1–2), 298–305. 10.1111/jocn.15520 33006794 PMC7537231

[jan15047-bib-0054] Hofmeyer, A. , Taylor, R. , & Kennedy, K. (2020a). Fostering compassion and reducing burnout: How can health system leaders respond in the Covid‐19 pandemic and beyond? Nurse Education Today, 94, 104502. 10.1016/j.nedt.2020.104502 32980180 PMC7295512

[jan15047-bib-0055] Hofmeyer, A. , Taylor, R. , & Kennedy, K. (2020b). Knowledge for nurses to better care for themselves so they can better care for others during the Covid‐19 pandemic and beyond. Nurse Education Today, 94, 104503. 10.1016/j.nedt.2020.104503 32980179 PMC7295457

[jan15047-bib-0056] Holmgren, J. , Paillard‐Borg, S. , Saaristo, P. , & von Strauss, E. (2019). Nurses’ experiences of health concerns, teamwork, leadership and knowledge transfer during an Ebola outbreak in West Africa. Nursing Open, 6(3), 824–833. 10.1002/nop2.258 31367405 PMC6650671

[jan15047-bib-0057] Humphreys, J. , Schoenherr, L. , Elia, G. , Saks, N. T. , Brown, C. , Barbour, S. , & Pantilat, S. Z. (2020). Rapid implementation of inpatient telepalliative medicine consultations during COVID‐19 pandemic. Journal of Pain & Symptom Management, 60(1), e54–e59. 10.1016/j.jpainsymman.2020.04.001 32283219 PMC7151239

[jan15047-bib-0058] Joo, J. Y. , & Liu, M. F. (2021). Nurses’ barriers to caring for patients with COVID‐19: A qualitative systematic review. International Nursing Review, 68, 202–213. 10.1111/inr.12648 33420749 PMC8013562

[jan15047-bib-0059] Kalisch, B. J. (2006). Missed nursing care: A qualitative study. Journal of Nursing Care Quality, 21, 306–313. 10.1097/00001786-200610000-00006 16985399

[jan15047-bib-0060] Kang, H. S. , Son, Y. D. , Chae, S. M. , & Corte, C. (2018). Working experiences of nurses during the Middle East respiratory syndrome outbreak. International Journal of Nursing Practice, 24(5), e12664. 10.1111/ijn.12664 29851209 PMC7165521

[jan15047-bib-0061] Kim, Y. (2018). Nurses’ experiences of care for patients with Middle East respiratory syndrome‐coronavirus in South Korea. American Journal of Infection Control, 46(7), 781–787. 10.1016/j.ajic.2018.01.012 29502886 PMC7132718

[jan15047-bib-0062] Kitson, A. , Conroy, T. , Wengstrom, Y. , Profetto‐McGrath, J. , & Robertson‐Malt, S. (2010). Defining the fundamentals of care. International Journal of Nursing Practice, 16, 423–434. 20649678 10.1111/j.1440-172X.2010.01861.x

[jan15047-bib-0063] Kuntz, J. G. , Kavalieratos, D. , Esper, G. J. , Ogbu, N. , Mitchell, J. , Ellis, C. M. , & Quest, T. (2020). Feasibility and acceptability of inpatient palliative care e‐family meetings during COVID‐19 pandemic. Journal of Pain & Symptom Management, 60(3), e28–e32. 10.1016/j.jpainsymman.2020.06.001 PMC7272163 32505643

[jan15047-bib-0064] Lee, J. Y. , Hong, J. H. , & Park, E. Y. (2020). Beyond the fear: Nurses’ experiences caring for patients with Middle East respiratory syndrome: A phenomenological study. Journal of Clinical Nursing, 29(17–18), 3349–3362. 10.1111/jocn.15366 32498126

[jan15047-bib-0065] Liu, H. , & Liehr, P. (2009). Instructive messages from Chinese nurses’ stories of caring for SARS patients. Journal of Clinical Nursing, 18(20), 2880–2887. 10.1111/j.1365-2702.2009.02857.x 19747256

[jan15047-bib-0066] Liu, Q. , Luo, D. , Haase, J. E. , Guo, Q. , Wang, X. Q. , Liu, S. , Xia, L. , Liu, Z. , Yang, J. , & Yang, B. X. (2020). The experiences of health‐care providers during the COVID‐19 crisis in China: A qualitative study. The Lancet Global Health, 8(6), e790–e798. 10.1016/S2214-109X(20)30204-7 32573443 PMC7190296

[jan15047-bib-0067] Liu, Y. E. , Zhai, Z. C. , Han, Y. H. , Liu, Y. L. , Liu, F. P. , & Hu, D. Y. (2020). Experiences of front‐line nurses combating coronavirus disease‐2019 in China: A qualitative analysis. Public Health Nursing, 37(5), 757–763. 10.1111/phn.12768 32677072 PMC7405388

[jan15047-bib-0068] Maben, J. , Taylor, C. , & Bridges, J. (2020). Guidance to support nurses’ psychological well‐being during Covid‐19 crisis. https://eprints.soton.ac.uk/439503/1/Guidance_to_support_nurses_psychological_well_being_during_Covid_19_crisis_14.04.2020.pdf

[jan15047-bib-0069] Maltby, H. , & Conroy, C. (2020). Coalition of vermont nurse and nurse practitioner leaders responds to the ANA‐VT survey on COVID‐19: Themes and recommendations. Vermont Nurse Connection, 23(3), 7.

[jan15047-bib-0070] Manzano García, G. , & & Ayala Calvo, J. C. (2021). The threat of COVID‐19 and its influence on nursing staff burnout. Journal of Advanced Nursing, 77(2), 832–844. 10.1111/jan.14642 33155716

[jan15047-bib-0071] Martland, A. M. , & Huffines, M. (2020). Surge priority planning COVID‐19: Critical care staffing and nursing considerations. Chest. https://www.chestnet.org/resources/surge‐priority‐planning‐covid‐19‐critical‐care‐staffing‐and‐nursing‐considerations

[jan15047-bib-0072] McArthur, A. , Klugarova, J. , Yan, H. , & Florescu, S. (2015). Innovations in the systematic review of text and opinion. International Journal of Evidence‐Based Healthcare, 13(3), 188–195. 10.1097/XEB.0000000000000060 26207851

[jan15047-bib-0073] Medical Research Council . (2008). Developing and evaluating complex interventions: New guidance. https://mrc.ukri.org/documents/pdf/complex‐interventions‐guidance/

[jan15047-bib-0074] Moher, D. , Liberati, A. , Tetzlaff, J. , Altman, D. G. , & Group, T. P . (2009). Preferred reporting items for systematic reviews and meta‐analyses: The PRISMA statement. BMJ, 339, 10.1136/bmj.b2535 PMC3090117 21603045

[jan15047-bib-0075] Morley, G. , Sese, D. , Rajendram, P. , & Horsburgh, C. C. (2020). Addressing caregiver moral distress during the COVID‐19 pandemic. Cleveland Clinic Journal of Medicine, 9, 9. 10.3949/ccjm.87a.ccc047 32518134

[jan15047-bib-0076] Murrells, T. , Robert, G. , Adams, M. , Morrow, E. , & Maben, J. (2013). Measuring relational aspects of hospital care in England with the ‘Patient Evaluation of Emotional Care during Hospitalisation’ (PEECH) survey questionnaire. British Medical Journal Open, 3(1), e002211. 10.1136/bmjopen-2012-002211 PMC3563120 23370012

[jan15047-bib-0077] Neville, T. H. (2020). COVID‐19: A time for creative compassion. Journal of Palliative Medicine, 23(7), 990–991. 10.1089/jpm.2020.0242 32380916

[jan15047-bib-0078] Newby, J. C. , Mabry, M. C. , Carlisle, B. A. , Olson, D. M. , & Lane, B. E. (2020). Reflections on nursing ingenuity during the COVID‐19 pandemic. Journal of Neuroscience Nursing, 52(5), E13–E16. 10.1097/JNN.0000000000000525 PMC7172973 32221059

[jan15047-bib-0079] Pahuja, M. , & Wojcikewych, D. (2020). Systems barriers to assessment and treatment of COVID‐19 positive patients at the end of life. Journal of Palliative Medicine, 24(2), 302–304. 10.1089/jpm.2020.0190 32384004

[jan15047-bib-0080] Pentecost, C. , Frost, J. , Sugg, H. V. R. , Hilli, A. , Goodwin, V. A. , & Richards, D. A. (2020). Patients’ and nurses’ experiences of fundamental nursing care: A systematic review and qualitative synthesis. Journal of Clinical Nursing, 29, 1858–1882. 10.1111/jocn.15082 31661591 PMC7319357

[jan15047-bib-0081] Pettis, J. (2020). Creativity and compassion: NICHE nurses address older adult’s care needs during the COVID ‐19 pandemic. Geriatric Nursing. 41(4), 509–510. 10.1016/j.gerinurse.2020.06.005

[jan15047-bib-0082] Rangachari, P. , & L. Woods, J. (2020). Preserving organizational resilience, patient safety, and staff retention during COVID‐19 requires a holistic consideration of the psychological safety of healthcare workers. International Journal of Environmental Research and Public Health, 17(12), 15. 10.3390/ijerph17124267 PMC7345925 32549273

[jan15047-bib-0083] Rathert, C. , Wyrwich, M. D. , & Boren, S. A. (2013). Patient‐centered care and outcomes: A systematic review of the literature. Medical Care Research and Review, 70, 351–379. 10.1177/1077558712465774 23169897

[jan15047-bib-0084] Registered Nurses Association of Ontario . (2003). SARS unmasked: Celebrating resilience, exposing vulnerability. A report on the nursing experience with SARS in Ontario. https://www.yumpu.com/en/document/read/52533573/sars‐unmasked‐celebrating‐resilience‐exposing‐vulnerability

[jan15047-bib-0085] Richards, D. A. , Hilli, A. , Pentecost, C. , Goodwin, V. A. , & Frost, J. (2018). Fundamental nursing care: A systematic review of the evidence on the effect of nursing care interventions for nutrition, elimination, mobility and hygiene. Journal of Clinical Nursing, 27, 2179–2188. 10.1111/jocn.14150 29156087 PMC6001513

[jan15047-bib-0086] Richards, D. A. , Sugg, H. V. R. , Cockcroft, E. , Cooper, J. , Cruickshank, S. , Doris, F. , & Romanczuk, L. (2021). COVID‐NURSE: Development and evaluation of the effects of a COVID‐specific fundamental nursing care protocol compared to care as usual on experience of care for non‐invasively ventilated patients in hospital with the SARS‐CoV‐2 virus: Protocol for a cluster randomised controlled trial. BMJ, 11(5). e046436. 10.1136/bmjopen-2020-046436 PMC8159671 34039574

[jan15047-bib-0087] Rosa, W. E. , Ferrell, B. R. , & Wiencek, C. (2020). Increasing critical care nurse engagement of palliative care during the COVID‐19 pandemic. Critical Care Nurse, 40(6), e28–e36. 10.4037/ccn2020946 32699889 PMC8034497

[jan15047-bib-0088] Sharma, S. K. , Nuttall, C. , Kalyani, V. , & Hemlata, H. (2020). Clinical nursing care guidance for management of patient with COVID‐19. Journal of the Pakistan Medical Association, 70(Suppl 3), S118–S123. 10.5455/JPMA.29 32515397

[jan15047-bib-0089] Shih, F. J. , Gau, M. L. , Kao, C. C. , Yang, C. Y. , Lin, Y. S. , Liao, Y. C. , & Sheu, S. J. (2007). Dying and caring on the edge: Taiwan's surviving nurses’ reflections on taking care of patients with severe acute respiratory syndrome. Applied Nursing Research, 20(4), 171–180. 10.1016/j.apnr.2006.08.007 17996803 PMC7127079

[jan15047-bib-0090] Shih, F. J. , Turale, S. , Lin, Y. S. , Gau, M. L. , Kao, C. C. , Yang, C. Y. , & Liao, Y. C. (2009). Surviving a life‐threatening crisis: Taiwan's nurse leaders’ reflections and difficulties fighting the SARS epidemic. Journal of Clinical Nursing, 18(24), 3391–3400. 10.1111/j.1365-2702.2008.02521.x 19207797

[jan15047-bib-0091] Suhonen, R. , Leino‐Kilpi, H. , & Valimaki, M. (2005). Development and psychometric properties of the Individualized Care Scale. Journal of Evaluation in Clinical Practice, 11(1), 7–20. 10.1111/j.1365-2753.2003.00481.x 15660532

[jan15047-bib-0092] Taylor, E. J. (2020). During the COVID‐19 pandemic, should nurses offer to pray with patients? Nursing, 50(7), 42–46. 10.1097/01.NURSE.0000668624.06487.72 32558790

[jan15047-bib-0093] Tiwari, A. , Chan, S. , Wong, A. , Tai, J. , Cheng, K. , Chan, J. , & Tsang, K. (2003). Severe acute respiratory syndrome (SARS) in Hong Kong: Patients’ experiences. Nursing Outlook, 51(5), 212–219. 10.1016/j.outlook.2003.07.002 14569227 PMC7125875

[jan15047-bib-0094] Tsai, M.‐J. , Tsai, W.‐T. , Pan, H.‐S. , Hu, C.‐K. , Chou, A.‐N. , Juang, S.‐F. , Huang, M.‐K. , & Hou, M.‐F. (2020). Deployment of information technology to facilitate patient care in the isolation ward during COVID‐19 pandemic. Journal of the American Medical Informatics Association, 27(11), 1819–1820. 10.1093/jamia/ocaa126 32516374 PMC7314018

[jan15047-bib-0095] Umoren, R. A. , Gray, M. M. , Handley, S. , Johnson, N. , Kunimura, C. , Mietzsch, U. , Billimoria, Z. , & Lo, M. D. (2020). In‐hospital telehealth supports care for neonatal patients in strict isolation. American Journal of Perinatology, 37(8), 857–860. 10.1055/s-0040-1709687 32268382 PMC7356060

[jan15047-bib-0096] Verbeek, J. H. , Rajamaki, B. , Ijaz, S. , Sauni, R. , Toomey, E. , Blackwood, B. , & Kilinc Balci, F. S. (2020). Personal protective equipment for preventing highly infectious diseases due to exposure to contaminated body fluids in healthcare staff. Cochrane Database Systematic Review, 5(CD0011621). 1–153. 10.1002/14651858.CD011621 PMC8785899 32412096

[jan15047-bib-0097] Vincent, C. , & Amalberti, R. (2016). Safer healthcare: Strategies for the real world. 29465922

[jan15047-bib-0098] Viswanathan, R. , Myers, M. F. , & Fanous, A. H. (2020). Support groups and individual mental health care via video conferencing for frontline clinicians during the COVID‐19 pandemic. Psychosomatics, 23, 23. 10.1016/j.psym.2020.06.014 PMC7308785 32660876

[jan15047-bib-0099] Wakam, G. K. , Montgomery, J. R. , Biesterveld, B. E. , & Brown, C. S. (2020). Not dying alone—Modern compassionate care in the Covid‐19 pandemic. The New England Journal of Medicine, 382(24), 10.1056/NEJMp2007781 PMC7768603 32289215

[jan15047-bib-0100] Wang, H. , Zeng, T. , Wu, X. , & Sun, H. (2020). Holistic care for patients with severe coronavirus disease 2019: An expert consensus. International Journal of Nursing Sciences, 7(2), 128–134. 10.1016/j.ijnss.2020.03.010 32292634 PMC7128660

[jan15047-bib-0101] Wang, X. , Sun, C. , Hu, H.‐X. , Wang, Z.‐X. , Wang, H. , Peng, H. , Qiao, J.‐H. , Gao, L. , Cai, D. , Cai, M. , Deng, C. , Deng, Y. , Han, Y. , Jiang, Y. , Li, H. , Liao, Y. , Wang, L. , Wu, X. , Yue, L. , … Zhou, T. (2020). Expert consensus on the nursing management of critically ill elderly patients with coronavirus disease 2019. Aging Medicine, 3(2), 74–81. 10.1002/agm2.12107 32666025 PMC7338692

[jan15047-bib-0103] Wilson, D. (2015). CE: Inside an Ebola Treatment Unit: A Nurse's Report. American Journal of Nursing, 115(12), 28–38. 10.1097/01.NAJ.0000475288.30664.70 26559159

[jan15047-bib-0102] World Health Organization . (2016). Guidelines on core components of infection prevention and control programmes at the national and acute health care facility level. https://www.who.int/gpsc/core‐components.pdf 27977095

