# Supplementary material for: Impact of COVID‐19 and other infectious conditions requiring isolation on the provision of and adaptations to fundamental nursing care in hospital in terms of overall patient experience, care quality, functional ability, and treatment outcomes: systematic review
Source: J Adv Nurs. 2021 Sep 23;78(1):78–108. doi: 10.1111/jan.15047 (PMC8657334; doi:10.1111/jan.15047)
Supplement: Supplementary file 1 — Appendix A [file JAN-78-78-s001.doc]

**Medline via OvidSP search strategy**

--------------------------------------------------------------------------------

1 ncov*.tw,kf. (1053)

2 2019ncov*.tw,kf. (9)

3 covid*.tw,kf. (32402)

4 sars.tw,kf. (19795)

5 sarscov*.tw,kf. (90)

6 Severe Acute Respiratory Syndrome*.tw,kf,ox. (18519)

7 coronavir*.tw,kf. (25694)

8 coronovir*.tw,kf. (24)

9 betacoronavir*.tw,kf. (370)

10 cov.tw,kf. (15683)

11 hcov*.tw,kf. (696)

12 ((Wuhan or Hubei) adj5 pneumonia).tw,kf. (205)

13 sudden acute respiratory syndrome*.tw,kf. (13)

14 mers.tw,kf. (4944)

15 Middle East Respiratory.tw,kf. (2289)

16 pandemic*.ti. (16839)

17 ebola*.ti. (6373)

18 "swine flu".tw,kf. (960)

19 h1n1.tw,kf. (17571)

20 exp Coronavirus/ or exp Coronavirus Infections/ (28421)

21 exp Betacoronavirus/ (17679)

22 *Influenza A Virus, H1N1 Subtype/ (11665)

23 1 or 2 or 3 or 4 or 5 or 6 or 7 or 8 or 9 or 10 or 11 or 12 or 13 or 14 or 15 or 16 or 17 or 18 or 19 or 20 or 21 or 22 (92866)

24 nurse$1.ti. (114556)

25 nursing*.ti. (149282)

26 nurse$1.tw,kf. (265677)

27 nursing.tw,kf. (269328)

28 *nurses/ or exp nurse practitioners/ or exp nurse specialists/ or exp nursing staff/ (127362)

29 *nursing/ or *evidence-based nursing/ or *nursing research/ or *specialties, nursing/ or *nutritional sciences/ (54181)

30 (protocol* or guideline* or guidance or care* or support* or barrier* or procedure* or respiration or breathing).tw,kf. (4853275)

31 24 or 25 (255676)

32 26 or 27 or 28 or 29 (520614)

33 30 and 32 (236615)

34 31 or 33 (395853)

**35 23 and 34 (1308) SEARCH 1**

36 (patient* adj2 (need* or care or safety or wellbeing or "well being")).ti. (40193)

37 patient care.ab. (55176)

38 ((compassion* or fundamental) adj2 care).ab. (1448)

39 (fundamental and care).ti. (197)

40 (inpatient* adj2 (need* or care or safety or wellbeing or "well being")).ti. (1795)

41 inpatient care.ab. (5155)

42 36 or 37 or 38 or 39 or 40 or 41 (100075)

43 23 and 42 (730) SEARCH 2

**44 35 or 43 (1951)**

45 dignity.ti. (2186)

46 compassion*.ti. (3463)

47 privacy.ti. (4384)

48 communicat*.ti. (64953)

49 comfort*.ti. (4865)

50 empath*.ti. (4928)

51 45 or 46 or 47 or 48 or 49 or 50 (84340)

52 23 and 51 (370)

53 32 and 52 (10) SEARCH 3

**54 44 or 53 (1951)**

55 nutrition*.ti. (101639)

56 malnutrition.ti. (11985)

57 undernutrition.ti. (2254)

58 fluid$1.ti. (121475)

59 hydrat*.ti. (15399)

60 diet*.ti. (189418)

61 continen*.ti. (8040)

62 incontinen*.ti. (22220)

63 catheter*.ti. (62604)

64 sleep*.ti. (96526)

65 hygien*.ti. (30079)

66 skin.ti. (156292)

67 bath.ti. (3111)

68 bathing.ti. (1434)

69 activit*.ti. (664138)

70 exercis*.ti. (119895)

71 mobility.ti. (22986)

72 oral health.ti. (11980)

73 or/55-72 (1596285)

74 23 and 73 (2784)

75 32 and 74 (36)

76 75 (36)

77 limit 76 to yr="2016 -Current" (22) SEARCH 4

***Explanation:***

***Limit due to previous systematic reviews having been done on these and the searches done in 2016.***

78 54 or 77 (1957)

79 exp Qualitative Research/ (55758)

80 theme*.tw. (90493)

81 qualitative*.tw. (272310)

82 79 or 80 or 81 (339590)

83 32 and 82 (35954)

84 23 and 83 (100) SEARCH 5

85 78 or 84 (1964)

***************************
