# Supplementary material for: Impact of COVID‐19 and other infectious conditions requiring isolation on the provision of and adaptations to fundamental nursing care in hospital in terms of overall patient experience, care quality, functional ability, and treatment outcomes: systematic review
Source: J Adv Nurs. 2021 Sep 23;78(1):78–108. doi: 10.1111/jan.15047 (PMC8657334; doi:10.1111/jan.15047)
Supplement: Supplementary file 2 — Appendix B [file JAN-78-78-s002.docx]

| ***Impact of SARS-CoV-2 (COVID-19) and other conditions requiring isolation on the provision of fundamental nursing care in hospital: systematic review*** | | | | | | | | |  |
| --- | --- | --- | --- | --- | --- | --- | --- | --- | --- |
|  |  | **Database searches (*date run: July 2020 date re-run: March 2021*)** | | | | | | |  |
| **Included references** | **Study type** | ***BNI*** | ***CINAHL*** | ***Medline*** | ***PsycINFO*** | ***Medrxiv/bioxiv*** | ***WHO covid database*** | ***google scholar*** | ***Suppl. searches*** |
| Adams 2020 | Opinion/ Editorial |  |  | z |  |  | x |  |  |
| Aguilla 2020 | Protocol |  |  | z |  |  | x |  |  |
| Anderson 2020 | Literature Review |  | x | z |  |  |  |  |  |
| Anderson 2020b | Literature Review | x |  | n |  |  |  |  |  |
| Andertun 2017 | Qualitative study |  |  | x |  |  |  |  |  |
| Bagnasco 2020 | Opinion/ Editorial |  |  | x |  |  |  |  |  |
| Bouchoucha 2020 | Opinion/ Editorial |  |  | x |  |  |  |  |  |
| Brown-Johnson 2020 | Opinion/ Editorial |  |  | x |  |  |  |  |  |
| Buheji 2020 | Protocol |  |  | x |  |  |  |  |  |
| Caccialanza 2020 | Protocol | x |  | z |  |  |  |  |  |
| Cathcart 2020 | Opinion/ Editorial | x |  | z |  |  |  |  |  |
| Cena 2020 | Protocol |  |  | n |  |  | x |  |  |
| Chan 2006 | Observational study |  |  | x |  |  |  |  |  |
| Chan 2006b | Observational study |  |  | x |  |  |  |  |  |
| Chan 2008 | Trial/ Cohort/ Case-Control |  |  | x |  |  |  |  |  |
| Chapple 2020 | Protocol |  |  | n |  |  | x |  |  |
| Cheng 2005 | Qualitative study |  | x | n |  |  |  |  |  |
| Chochinov, 2020 | Opinion/ Editorial |  |  | z |  |  | x |  |  |
| Cintoni, 2020 | Opinion/ Editorial | x |  | z |  |  |  |  |  |
| Corley 2010 | Qualitative study |  |  | x |  |  |  |  |  |
| Danielis 2020 | Opinion/ Editorial |  |  | x |  |  |  |  |  |
| De Lima 2020 | Protocol |  |  | x |  |  |  |  |  |
| DeCastro 2020 | Opinion/ Editorial |  |  | n |  |  |  | x |  |
| Diamond 2020 | Opinion/ Editorial |  |  | x |  |  |  |  |  |
| Dingfield 2020 | Opinion/ Editorial |  |  | z |  |  | x |  |  |
| Estella 2020 | Opinion/ Editorial |  |  | x |  |  |  |  |  |
| Fan 2020 | Opinion/ Editorial |  |  | x |  |  |  |  |  |
| Fang 2020 | Opinion/Editorial |  | x | y |  |  |  |  |  |
| Fausto 2020 | Protocol |  |  | x |  |  |  |  |  |
| Fedele 2020 | Opinion/ Editorial |  |  | n |  |  |  | x |  |
| Feder 2020 | Opinion/ Editorial |  |  | z |  |  | x |  |  |
| Hart 2020 | Opinion/ Editorial |  |  | x |  |  |  |  |  |
| Hofmeyer 2020 a | Opinion/ Editorial |  |  | y |  |  | x |  |  |
| Hofmeyer 2020 b | Opinion/ Editorial |  |  | y |  |  | x |  |  |
| Holmgren 2019 | Mixed methods study |  |  | x |  |  |  |  |  |
| Humphreys 2020 | Opinion/ Editorial |  |  | x |  |  |  |  |  |
| Kang 2018 | Qualitative study |  |  | x |  |  |  |  |  |
| Kim 2018 | Qualitative study |  |  | x |  |  |  |  |  |
| Kuntz 2020 | Mixed methods study |  |  | x |  |  |  |  |  |
| Lee 2020 | Qualitative study |  |  | x |  |  |  |  |  |
| Liu,H 2009 | Qualitative study |  |  | x |  |  |  |  |  |
| Liu,Q 2020 | Qualitative study |  |  | x |  |  |  |  |  |
| Liu,Y 2020 | Qualitative study |  |  | x |  |  |  |  |  |
| Maben 2020 | Protocol |  |  | n |  |  |  | x |  |
| Maltby 2020 | Opinion/ Editorial |  | x | n |  |  |  |  |  |
| Martland 2020 | Opinion/ Editorial |  |  | n |  |  |  | x |  |
| Morely 2020 | Consensus Statement |  |  | x |  |  |  |  |  |
| Neville 2020 | Opinion/ Editorial |  |  | z |  |  | x |  |  |
| Newby 2020 | Opinion/ Editorial |  |  | x |  |  |  |  |  |
| Nursing Department 2020 | Consensus Statement |  |  | x |  |  |  |  |  |
| Pahuja 2020 | Opinion/ Editorial |  |  | x |  |  |  |  |  |
| Pettis 2020 | Opinion/ Editorial |  |  | n |  |  | x |  |  |
| Ranjachari 2020 | Opinion/ Editorial |  |  | x |  |  |  |  |  |
| Rosa 2020 | Literature review |  |  | x |  |  |  |  |  |
| Sharma 2020 | Literature review |  |  | x |  |  |  |  |  |
| Shih 2007 | Qualitative study |  |  | x |  |  |  |  |  |
| Shih 2009 | Qualitative study |  |  | x |  |  |  |  |  |
| Taylor 2020 | Opinion/ Editorial |  |  | x |  |  |  |  |  |
| Tiwari 2003 | Qualitative study |  |  | x |  |  |  |  |  |
| Tsai 2020 | Opinion/ Editorial |  |  | x |  |  |  |  |  |
| Umoren 2020 | Observational study |  | x | z |  |  |  |  |  |
| Viswanathan 2020 | Observational study |  |  | x |  |  |  |  |  |
| Wakam 2020 | Opinion/ Editorial | x |  | z |  |  |  |  |  |
| Wang 2020 | Consensus statement |  |  | x |  |  |  |  |  |
| No. included refs |  | 5 | 5 | 39 | 0 | 0 | 11 | 4 |  |
| Yield |  | 527 | 720 | 1964 | 203 | 25 | 809 | 269 |  |
| No. database searches carried out = | | | 7 |  |  |  |  |  |  |
| Sum of yields = | | | 4517 |  | NNR | | | 48.21875 |  |
| No. refs screened at Ti&Ab = | | | 3086 |  | NNR FT | | | 2.609375 |  |
| No. refs screened at FT= | | | 167 |  | NNS | | | 18.479042 |  |
| No. of included refs from searching = | | | 64 |  | Overall Sensitivity | | | 100 |  |
| Total no. of included refs = | | | 64 |  | Overall precision | | | 2.073882 |  |
|  |  |  |  |  |  |  |  |  |  |
| **Codes** |  |  |  |  | **Other codes** | |  |  |  |
| x = found from the search | | | |  | NNR = number needed to read. 1/overall precision | | | |  |
| y = in database; found when search strategy re-run | | | |  | NNR FT = number needed to read at FT to find one included reference | | | | |
| n = not in the database | | | |  | NNS = number needed to screen to find one reference to include for FT screening | | | | |
| z = in the database; not found using the search strategy | | | |  |  |  |  |  |  |
| (red) **=** database where searches re-run | | | |  |  |  |  |  |  |
